# Opposite Effects of M1 and M2 Macrophage Subtypes on Lung Cancer Progression

**DOI:** 10.1038/srep14273

**Published:** 2015-09-24

**Authors:** Ang Yuan, Yi-Jing Hsiao, Hsuan-Yu Chen, Huei-Wen Chen, Chao-Chi Ho, Yu-Yun Chen, Yi-Chia Liu, Tsai-Hsia Hong, Sung-Liang Yu, Jeremy J.W. Chen, Pan-Chyr Yang

**Affiliations:** 1 Departments of Chest Medicine and Emergency Medicine, National Taiwan University Hospital, Taipei, Taiwan; 2 Department of Clinical Laboratory Sciences and Medical Biotechnology, National Taiwan University College of Medicine, Taipei, Taiwan; 3 Institute of Statistical Science, Academia Sinica, Taipei, Taiwan; 4 Graduate Institute of Toxicology, National Taiwan University College of Medicine, Taipei, Taiwan; 5 Department of Internal Medicine, National Taiwan University College of Medicine, Taipei, Taiwan; 6 Departments of Surgery, National Taiwan University Hospital, Taipei, Taiwan; 7 General Education Center, National Defense University, Taipei, Taiwan; 8 Department of Laboratory Medicine, National Taiwan University Hospital, Taipei, Taiwan; 9 Department of Pathology, National Taiwan University College of Medicine, Taipei, Taiwan; 10 Center for Optoelectronic Biomedicine, National Taiwan University College of Medicine, Taipei, Taiwan; 11 Institute of Biomedical Sciences, National Chung-Hsing University, Taichung, Taiwan; 12 Agricultural Biotechnology Center, National Chung-Hsing University, Taichung, Taiwan

## Abstract

Macrophages in a tumor microenvironment have been characterized as M1- and M2-polarized subtypes. Here, we discovered the different macrophages’ impacts on lung cancer cell A549. The M2a/M2c subtypes promoted A549 invasion and xenograft tumor growth. The M1 subtype suppressed angiogenesis. M1 enhanced the sensitivity of A549 to cisplatin and decreased the tube formation activity and cell viability of A549 cells by inducing apoptosis and senescence. Different macrophage subtypes regulated genes involved in the immune response, cytoskeletal remodeling, coagulation, cell adhesion, and apoptosis pathways in A549 cells, which was a pattern that correlated with the altered behaviors of the A549 cells. Furthermore, we found that the identified M1/M2 gene signatures were significantly correlated with the extended overall survival of lung cancer patients. These results suggest that M1/M2 gene expression signature may be used as a prognostic indicator for lung cancer patients, and M1/M2 polarization may be a target of investigation of immune-modulating therapies for lung cancer in the future.

The tumor microenvironment has been an issue of long-standing importance in tumor biology. Many stromal cells, through interacting with tumor cells, modify the tumor stroma and ultimately promote angiogenesis and tumor metastasis^1^,^2^. Inflammation, a hallmark of cancer that has been studied since 1980, supplies tumor cells with sufficient growth factors or matrix-degrading enzymes that are important for their survival, metastasis and angiogenesis^3^. Inflammatory cells, especially tumor-associated macrophages (TAMs), are recruited by tumor cells and infiltrate tumor tissues^4^,^5^.

Recent investigations have shown that TAMs can promote tumor development and progression by promoting angiogenesis, matrix remodeling and suppressing adaptive immunity^6^,^7^. Although numerous clinical studies have shown that the TAM count in tumors is correlated with poor-patient prognosis in many cancers^6^, a few studies have presented contrary results^8^,^9^,^10^. Our previous studies showed that TAMs are associated with angiogenesis and correlate with poor-patient survival from non-small cell lung cancer (NSCLC)^11^. However, other investigations have yielded conflicting results, showing that NSCLC patient survival is extended if tumor islets are infiltrated with more macrophages^9^,^12^. It may be that differences in TAM locations or the activation of macrophage subsets may differentially affect the diverse functions of TAMs in tumor progression.

Previous studies have shown that interactions with macrophages can enhance the invasiveness and matrix-degrading activity of cancer cells^6^, and macrophages have been shown to alter gene expression profiles in lung cancer cell lines after co-culturing^11^. These results indicate that TAMs may exert important effects on lung cancer cells by modulating their biological behaviors and regulating their global gene expression pattern.

Recently, macrophages were classified as M1 and M2 subtypes depending on the immune response that was induced, a Th1 or Th2 response^4^,^13^. Mantovani and his colleagues further classified macrophages into M1, M2a, M2b and M2c based on the cytokines and immune functions that were produced^14^. Several recent studies have shown that M1 and M2 macrophages were distributed throughout human cancer tissues^15^,^16^. However, the exact effects of different TAM subtypes, such as M1 versus the different M2 subtypes, on the regulation of gene expression and modulation of the biological behaviors of lung cancer cells have not been fully elucidated. Particularly, whether expression pattern of cancer cells induced by different TAM subtypes is associated with patients’ outcome is never reported, to the best of our knowledge. In this report, we evaluated the changes in the biological behaviors and then determined the global gene expression profile of NSCLC cells after co-culturing with different macrophage subtypes. Finally, we calculated the M1/M2 gene signatures and correlated these signatures with the prognosis of patients with lung cancer.

## Results

### Polarization of macrophages into different subtypes

**Figure 1:**
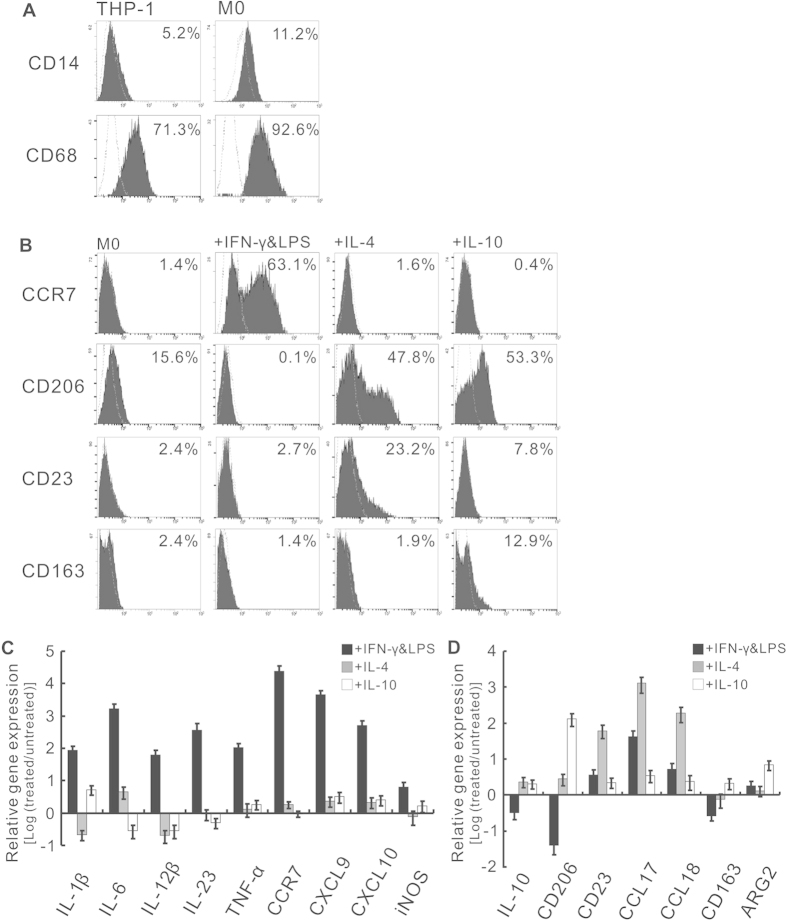
Cytokine-induced macrophage polarization. (**A**) M0 macrophage differentiation from monocytic THP-1 cells. The M0 macrophages were characterized by flow cytometry with CD68 staining. Dotted lines indicated the isotype controls. (**B**) Validation of M1 and M2a/c polarization by the flow cytometry. CCR7, CD23 and CD163 are specific surface markers of M1, M2a and M2c macrophages, respectively, and the marker CD206 is common to both M2 subtypes. Dotted lines indicated the isotype controls. (**C**) Expression of Th1 cytokines and M1 markers in differentiated macrophage subtypes, determined by real-time RT-PCR. (**D**) Expression of Th2 cytokines and M2 markers in differentiated macrophage subtypes, determined by real-time RT-PCR. The gene expression of differentiated macrophage subtypes was normalized to that of untreated M0 macrophages. The scale is the base 10 logarithm of the ratio of the relative expression of gene in cytokine-treated M0 to that in untreated M0. The log ratio greater than 0 is considered as upregulation compared with M0, and vice versa. Experiments in (**C**) and (**D**) were performed in triplicate, respectively.

Flow cytometry analysis showed that the expression of CD14 and CD68 was up-regulated in M0 macrophages compared to THP-1 cells, although the basal CD68 expression was high in THP-1 cells (Fig. 1A). CCR7 was almost exclusively expressed in M1 macrophages (Fig. 1B), whereas CD206 was more highly expressed in M2a and M2c macrophages, which was consistent with a previous report^17^. Additionally, CD23 was more highly expressed in the M2a subtype, and CD163 was more highly expressed in the M2c subtype. To further characterize the macrophage subtypes, we measured the expression of cytokines that are commonly responsible for the Th1 and Th2 responses in polarized macrophages by real-time RT-PCR. The Th1 cytokines IL-1, IL-6, TNF-α and IL-23 were up-regulated in M1 macrophages, whereas the Th2 cytokine IL-10 was up-regulated in M2 macrophages (Fig. 1C,D).

### M1 macrophages decrease the viability and proliferation of A549 cells and enhance their drug sensitivity

**Figure 2:**
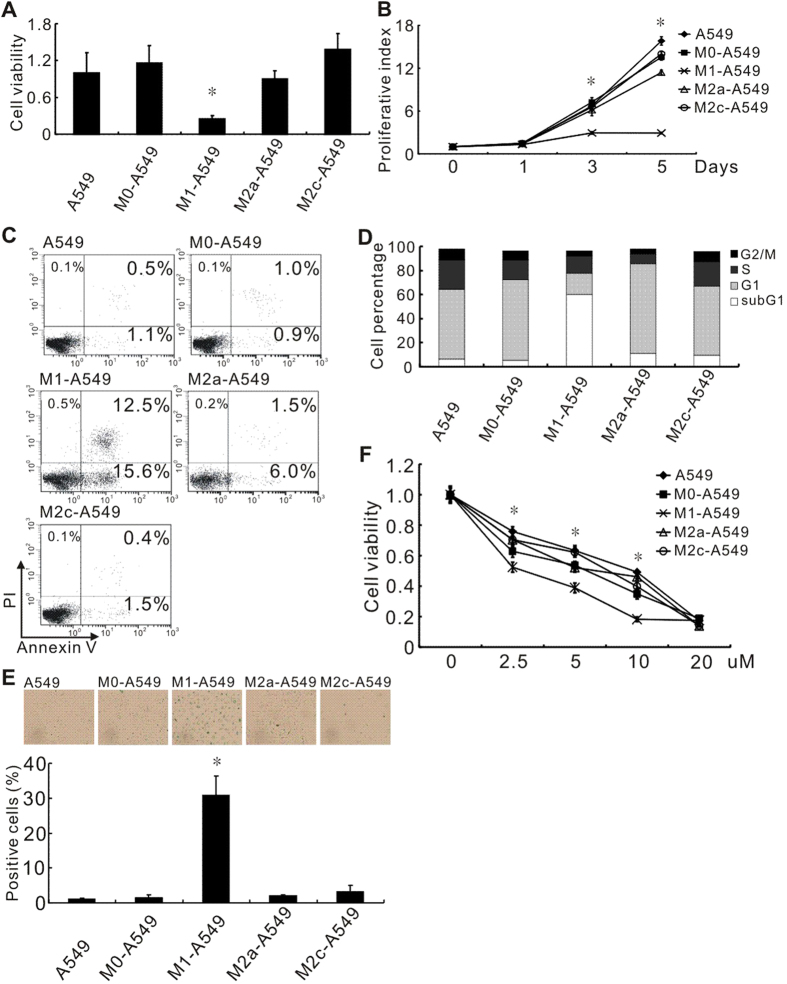
M1 macrophage CM reducing cell viability and enhancing drug sensitivity of lung cancer cells. (**A**) Evaluation of A549 lung cancer cell viability after culturing with macrophage CM for 5 days by counting cells. *P < 0.05 (mean ± SD, n = 3). Experiments were performed in three independent triplicates. Each value of bar is presented as the average of 9 assays. (**B**) Proliferation of long-term-cultured A549 cells, determined by the MTT assay. *P < 0.05 (mean ± SD, n = 3) Experiments were performed in triplicate. (**C**) Apoptosis of A549 cells after treatment with macrophage CM for 5 days. Cell apoptosis was determined by flow cytometry with annexin V/PI-staining. Data were confirmed in three independent experiments. (**D**) Cell cycle distribution of CM-treated A549 cells determined by flow cytometry with PI-staining. The sub-G1 population corresponds to apoptotic cells.(**E**) Cellular senescence of CM-treated A549 cells assessed by counting β-galactosidase-positive cells. *P < 0.05 (mean ± SD, n = 3). Experiments were performed in triplicate. (**F**) The impact of macrophage subtypes on drug responsiveness. Long-term–cultured A549 cells were treated with the indicated concentrations of cisplatin. *P < 0.05 (mean ± SD, n = 3), compared with the M0 treatment. Experiments were performed in triplicate.

After CM (conditioned medium) treatment, only M1 macrophage CM significantly decreased A549 cell viability and proliferation as well as invasion ability compared with M0 macrophage CM as determined by counting the cell number, MTT and Boyden chamber assays, respectively (Fig. 2A,B and Supplementary Fig. 1). To further understand if the reduced cell number of M1 CM-treated cells was caused by cell growth retardation or cell death, we measured the apoptosis and senescence of CM-cultured cells by annexin V/PI staining and β-galactosidase staining, respectively. Annexin V/PI staining revealed that M1 CM co-culturing for 5 days induced A549 cell apoptosis (28.1%) (Fig. 2C). Upon long-term-co-culturing, cell-cycle analysis showed that M1 CM induced a dramatic sub-G1 accumulation of treated A549 cells (64.2%) when compared to other CMs (Fig. 2D). Moreover, 30% of viable long-term M1 CM-treated cells was β-galactosidase positive, whereas less than 5% of other CM-treated cells was β-galactosidase positive (Fig. 2E). These results indicated that apoptosis and senescence contribute to the anti-tumor effect of M1 macrophages. Because cisplatin can interact with DNA and cause apoptosis and cell-cycle arrest, this first-line chemotherapy drug for NSCLC was employed to treat A549 cells in long-term cultures with CM. The result revealed that M1 CM increased the cisplatin sensitivity of A549 cells compared with M0 CM (Fig. 2F).

### M1 macrophages suppress the tumorigenicity of A549 cells

**Figure 3:**
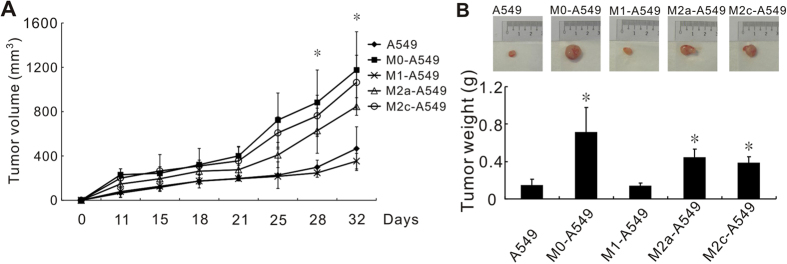
M1 macrophage CM reducing tumor growth *in vivo*. NOD-SCID mice were subcutaneously injected with long-term macrophage-co-cultured A549 cells. M1 subtype macrophages reduced xenograft tumor volume (**A**) and weight (**B**). *P < 0.05, compared with M0 macrophages. Each group contained six mice, and the data represent means ± standard deviations.

Because inflammation is important for tumor progression, we evaluated the inflammatory responses of lung cancer cells in 2-week co-culture conditions. To assess the effects of different polarized macrophages on tumorigenesis, long-term, macrophage-treated A549 cells which were counted by trypan blue to exclude the dead cells were subcutaneously injected into NOD/SCID mice. Agreeing with previous studies, our result showed that M0 and M2 co-culturing promoted tumor growth by significant increase in tumor volume and tumor weight compared with the A549 mock control^18^,^19^,^20^. In contrast, M1 co-culturing suppressed tumor growth compared with M0-treated A549 cells (Fig. 3A,B).

### M1 macrophages decrease A549 cell-induced angiogenesis *in vitro* and *in vivo*

**Figure 4:**
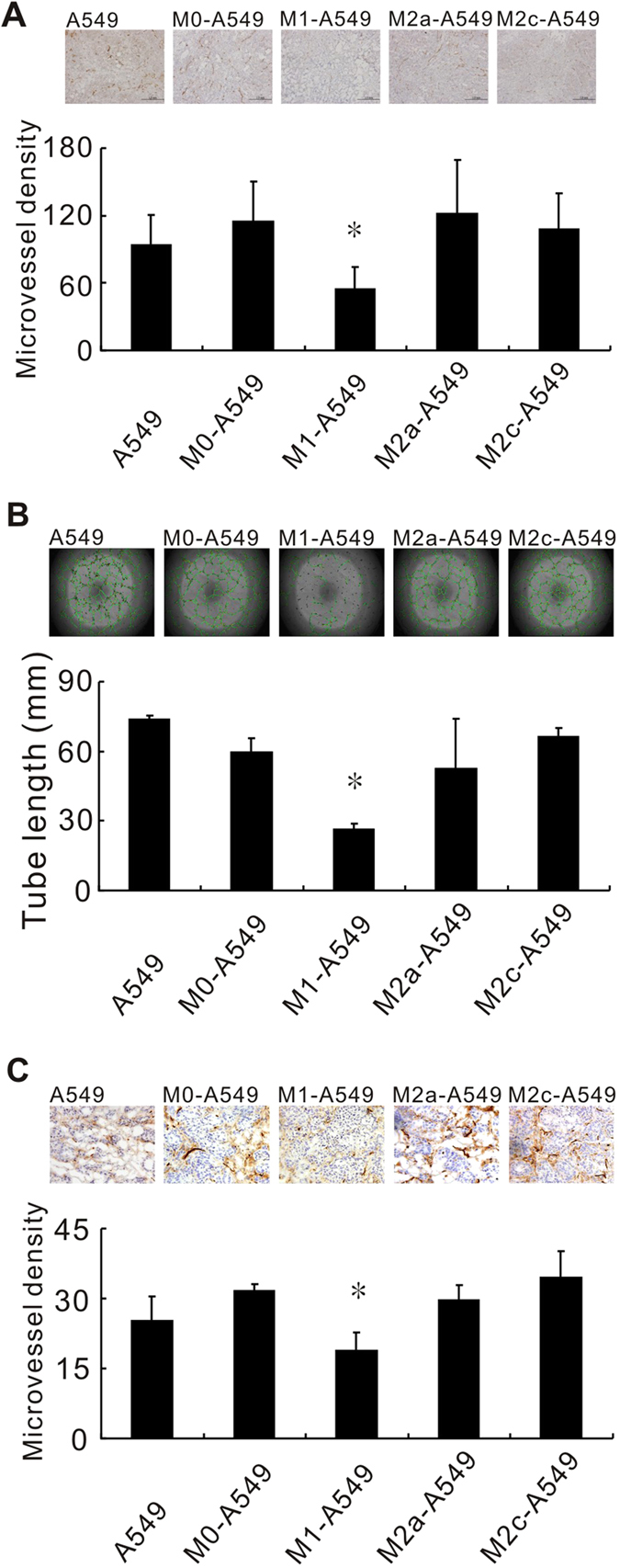
M1 macrophage reducing tumor angiogenesis. (**A**) Decreased microvessel density in xenograft tumors derived from M1-treated A549 cells, determined by immunohistochemical detection of the endothelial cell marker CD31. *P < 0.05 (mean ± SD, n = 3), compared to the M0-treated groups. The microvessel density for each group represents the mean value of three individual immunohistochemistry slides from three different mice. Each slide represents the average value of microvessel numbers of five fields. (**B**) *In vitro* tube formation. HUVECs were incubated with the CM of long-term macrophage-co-cultured A549 cells. *P < 0.05 (mean ± SD, n = 3), compared to the M0-treated group. Experiments were performed in triplicate. (**C**) *In vivo* angiogenesis, as determined by Matrigel plug assays. A mixture of Matrigel, A549 cells, and polarized macrophages was co-injected into NOD-SCID mice. Microvessels in the gel plugs were detected using the anti-CD31 antibody. *P < 0.05 (mean ± SD, n = 3), compared to the M0 -treated group.

In cancer progression, angiogenesis, which can be promoted by TAMs, is an indicator of tumor malignancy. We found that the microvessel density of tumors derived from NOD/SCID mice that were subcutaneously inoculated with M1 macrophage pretreated-A549 cells was lower when compared with M0 macrophage -co-cultured A549 cells (Fig. 4A). Next, we employed *in vitro* and *in vivo* assays to further clarify the effects of the different polarized macrophages on cancer cell-induced angiogenesis. *In vitro* tube-formation assays revealed that M1 macrophages significantly decreased A549-induced tube formation compared with M0 macrophages (Fig. 4B). Furthermore, *in vivo* Matrigel plug assays showed that plugs from M1 macrophage-treated A549 cells had fewer microvessels than those from M0 macrophage group (Fig. 4C).

### Oligonucleotide microarray and pathway analysis

**Figure 5:**
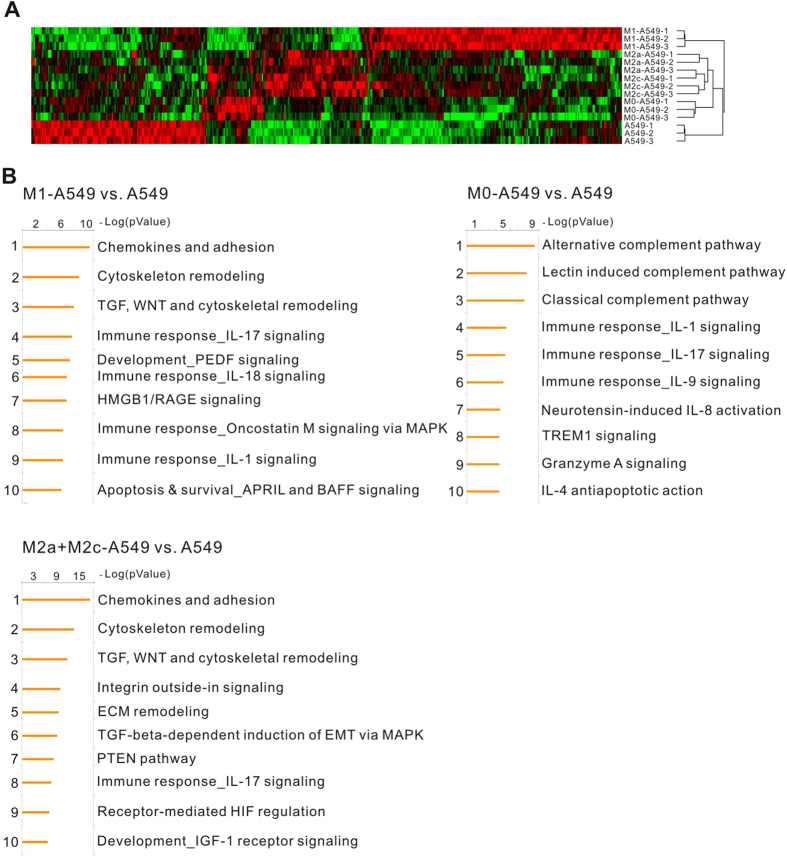
Gene expression profiles of A549 cells treated with polarized macrophages. (**A**) Heat map of differentially expressed genes in macrophage-treated A549 cells. A549 cells were cultured alone or co-cultured with M0, M1, M2a and M2c macrophages in Transwell plates for 48 hours, and the mRNAs were extracted for microarray analyses. All microarray experiments were performed in triplicate. (**B**) Pathway analysis of macrophage-altered genes. The top ten pathways were determined from the differentially expressed genes with equal or greater than 2-fold change between macrophage subtype-treated A549 cells and A549 mock control by Metacore software.

To investigate the underlying mechanism by which macrophages interfere with cancer behaviors, the transcriptomic profiles of A549 cells that were co-cultured with different macrophage subtypes were analyzed using microarrays in triplicate. The microarray data were quantile-normalized and filtered by an ANOVA (Analysis of Variance) under FDR (False Discovery Rate) protection (FDR <0.05). 497 genes were identified and subjected to hierarchical clustering analysis. Heat maps showed that the gene expression profiles of M2a-A549 and M2c-A549 cells were very similar and that the M0-A549 profile was closer to that of M2a/M2c-A549 (Fig. 5A). However, the patterns of A549 and M1-A549 cells were obviously different. Based on the selection criteria of a 2-fold change between the compared groups, 1096, 462 and 756 genes were selected from M1-A549, M0-A549 and M2a + M2c-A549, respectively, and compared to A549 cells alone. The genes identified above were then subjected to MetaCore pathway analysis (Fig. 5B). An example of the resulting data suggests that M1-treatment mainly affected cell-adhesion, Wnt-signaling pathways, immune response and apoptosis in A459 cells. The significantly differentially expressed genes in short- and long-term-cultured groups were further validated by real-time RT-PCR and categorized by biological functions (Supplementary Table 2). In general, the expression trends between short- and long-term co-cultured groups were similar, although the latter treatment paradigm had a stronger effect on gene expression.

### M1/M2 gene signatures associate with the survival of NSCLC patients

**Figure 6:**
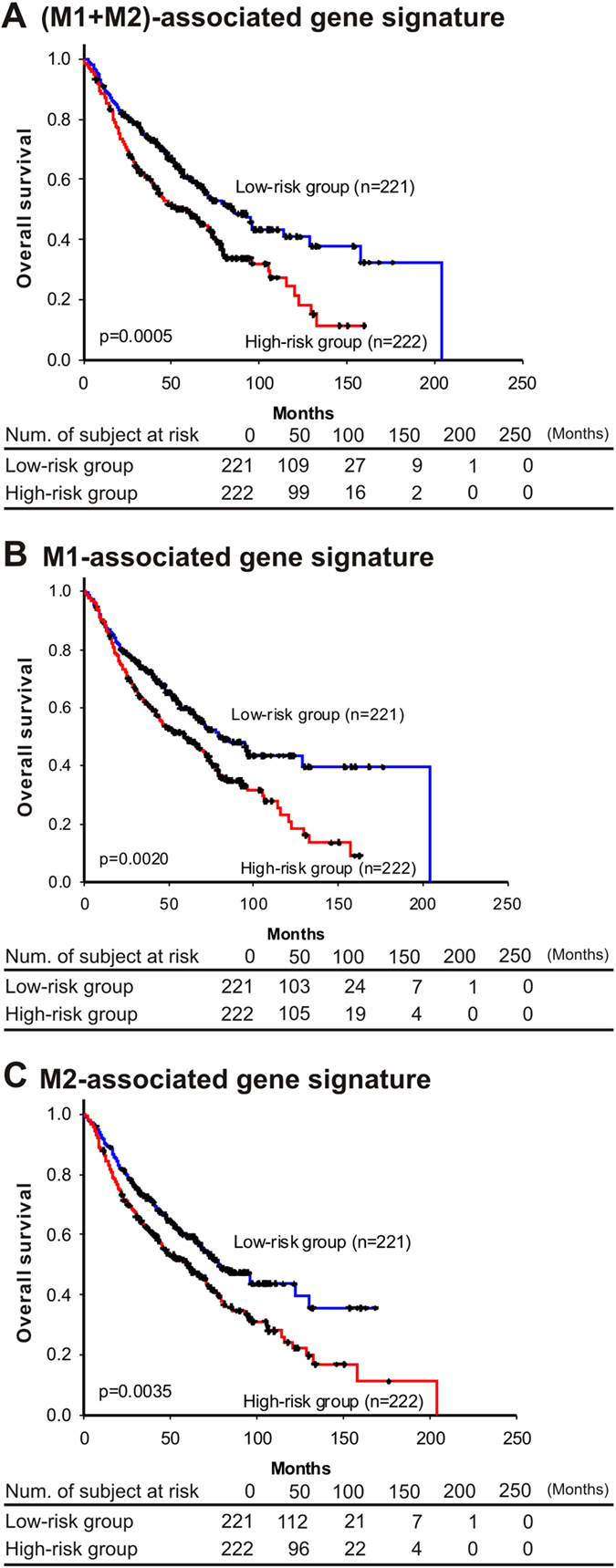
Kaplan–Meier estimates of NSCLC patient survival according to M1/M2 gene signatures. Overall survival curves were categorized based on the gene signatures of A549 lung cancer cells that were stimulated by macrophages of different subtypes. (**A**) M1/M2 combination gene signature derived from the union of the differentially expressed genes of M1-A549 and M2a/M2c-A549. (**B**) M1-specific gene signature derived from the differentially expressed genes of M1-A549 and unstimulated A549. (**C**) M2-specific gene signature derived from the differentially expressed genes of M2a/M2c-A549 and unstimulated A549. The datasets were obtained from UM, HLM, CAN/DF and MSK. The survival curve was estimated by the Kaplan-Meier method, and the log-rank test was performed to test the difference between the survival curves.

The results illustrated in Figs 2 and 3 revealed that M1 macrophages have anti-tumorigenic functions, and M2 macrophages exert a pro-tumorigenic effect. Thus, by using genes that were differentially expressed in M1 and M2 macrophage-stimulated A549 cells, we sought to identify the M1/M2 gene signatures to predict the clinical outcomes of NSCLC patients. Three gene signatures were identified based on the patient cohorts that had been published previously^21^, including an M1-specific gene signature (13 probes from 10 genes), M2-specific gene signature (3 probes from 3 genes) and M1/M2 combination gene signature (13 probes from 10 genes). Detailed information on the probes, genes and risk score formula for each gene signature is described in Supplementary Table 3. Indeed, the three derived risk scores could predict patient outcomes. Taking M1/M2 combination gene signature as an example, patients with a high-risk gene signature exhibited shorter, median overall survival than patients with a low-risk gene signature (*P* = 0.0005, log rank test; Fig. 6A). Similarly, the M1 and M2 gene signatures also predicted patient outcome (*P* = 0.0020, Fig. 6B; *P* = 0.0035, Fig. 6C, respectively). A multivariate Cox proportional hazards regression with covariates of age, gender and stage was used to evaluate the independent prognostic factors in the published cohort (n = 443). All hazard ratios of the gene signatures were still significant after considering the effects of covariates (Table 1). The results indicated that gene signatures from M1/M2 gene expression profiles correlated with extended overall survival in this published cohort.

## Discussion

TAMs in tumor microenvironment are associated with metastasis, angiogenesis and immunosuppression in various cancers^5^,^22^,^23^. Different macrophage subtypes, M1/M2, can serve as biomarkers for treatment and diagnosis^24^, and mouse studies suggest that macrophage polarization could serve as anti-cancer and anti-angiogenic therapeutic strategies^25^. In this study, we further demonstrated that macrophages with different polarizations differentially affect cancerous phenotypes and gene expression profiles of lung cancer cells. Furthermore, polarization-associated signatures can predict NSCLC patient survival.

Clinical data indicated that approximately 70% of TAMs were M2 macrophages and the remainder was M1 macrophages. Polarization of M1 into M2 occurred during cancer progression^15^,^26^, and the initial macrophages entering the tumor site were M1 and could be induced by innate immunity (IFN-γ)^26^,^27^. In our study, although less than 100% of the cells that were polarized *in vitro* expressed the corresponding surface markers (Fig. 1A,B), the pattern of cytokines was validated (Fig. 1C,D), confirming the specific macrophage subtypes and showing that the corresponding cytokine species were present in CM.

M2 macrophages have been considered to exert a tumor-promoting influence^28^. In basal cell carcinoma and breast cancer, M2 macrophages have been reported to mediate angiogenesis by inducing or releasing pro-angiogenic factors^7^,^29^. In our study, M2a and M2c subtypes could enhance cell invasion and tumor growth compared with A549 cells (Fig. 3 and Supplementary Fig. 1) but not angiogenesis (Fig. 4). These different effects might be attributed to different cancer cell types or the microenvironments in distinct organs. Furthermore, certain differentially expressed genes might contribute to invasion ability, such as fibrinogens (FGA, FGB and FGG)^30^ and tumor growth factor (TGF)-β^31^. Interestingly, the expression of these genes in cancer cells was profoundly decreased after interaction with M1 macrophages (Supplementary Table 2).

Regarding to the comparison of M2 and M0, our data agreed with the published data^18^,^19^,^20^. In previous reports, M0 or/and M2 promoted tumorigenesis in human-original and mouse-original lung cancer models. Although many studies indicated that TAMs might induce stemness, angiogenesis and lymphangiogenesis as well as drug resistance resulting in tumor promotion, the underlying mechanism of tumor associated macrophages involved in tumor progression is not understood thoroughly yet. However, these tumor-promoting effects are hard to be measured in the proliferation assay *in vitro*. Moreover, the percentages of apoptosis and senescence in the A549 cells without any stress were less than 5%. It is hard to demonstrate the tumor promoting activity of M0/M2 in these assay conditions. These facts might explain at least partly why M0 and M2 macrophages promoted tumor growth *in vivo* but showed no effects on the cancer cells *in vitro* compared with the mock control, A549 cells.

Hierarchical clustering analysis indicated that the closest relationship was between the M2a-A549 and M2c-A549 groups, which was followed by a second cluster of M2a/M2c-A549 and M0-A549 (Fig. 5A) that clearly elucidated the reason that M2a, M2c and M0 have similar effects on the biological functions of cancer cells. In our previous studies, PMA-activating M0 macrophages could enhance cancer cell invasiveness^11^,^23^. In agreement with these previous findings, we found that M0-A549 cell interaction produced larger tumor volumes *in vivo* than M1-A549 interactions (Fig. 3C). In this mouse experiment, A549 cells were injected after long-term culturing in CM of different macrophages, and the effects of M2 CM on A549 cells were maintained in NOD/SCID mice for at least 1 month (Fig. 3C), implying that the effects of inflammation on tumor cells persist for an extended period, even after TAMs (M2) are eliminated by anti-inflammatory drugs. On the other hand, M1-A549 suppressed tumor growth *in vivo* compared to M0-A549 cells and it had lower angiogenesis capacity (Figs 3 and 4A). This indicates the M1-mediated tumor suppression is attributed to the decrease of angiogenesis induced by M1 macrophages at least partly.

Our data showed that M0 and M2 macrophages increase cancer invasion ability (Supplementary Fig. 1), but M1 macrophages contribute to the suppression of tumor growth and angiogenesis (Figs 3 and 4) and enhance their sensitivity to chemotherapy agents (Fig. 2F). Notably, angiogenin (ANG), a therapeutic target that promotes tumor cell growth and angiogenesis in prostate and lung cancers^32^,^33^, was down-regulated in A549 cells by M1 under short-term and long-term culture conditions (Supplementary Table 2). ATF3 (activating transcription factor 3), a M1-up-regulated gene (Supplementary Table 2), is a transcription factor involved in the cellular response to cisplatin and control of the cell cycle^34^,^35^. IFI27 (interferon α-inducible protein 27) also enhances cisplatin sensitivity in head and neck squamous cell carcinomas^36^. The DNA damage-induced proteins, GADD34 and GADD153, respond to several cellular stresses and are regulated by members of the ATF family^37^,^38^,^39^. GADD45 induces apoptosis or cell-cycle arrest as part of the DNA damage-repair process^40^,^41^. Up-regulation of these genes by M1 macrophages is consistent with our data showing that M1-A549 cells were more sensitive to cisplatin, apoptosis and senescence (Fig. 2). Although the tumor-promoting cytokines IL-6 and IL-8 were also overexpressed in M1-A549 cells, it has been reported that these cytokines were produced by cells undergoing oncogene- or drug-induced senescence^42^,^43^. We found certain genes involved in the immune response, cytoskeletal remodeling, coagulation, cell adhesion and apoptosis pathways in macrophage-treated A549 cells based on microarray analysis. The underlying mechanism still remains further investigation.

In summary, our results show that M2a and M2c macrophages promote lung cancer cell invasion and tumor growth; in contrast, M1 macrophages suppress proliferation and cell viability of A549 cells, reduce angiogenesis *in vitro* and *in vivo*, increase the chemosensitivity of lung cancer cells, and induce the apoptosis and senescence of lung cancer cells. Different macrophage subtypes regulate genes involved in the immune response, cytoskeletal remodeling, coagulation, cell adhesion, and apoptosis pathways in A549 cells, which was a pattern that correlated with the altered behaviors of the A549 cells. In addition, the identified M1/M2 gene signatures were significantly correlated with the extended overall survival of lung cancer patients. To the best of our knowledge, this is the first report to demonstrate that M1/M2 gene signatures correlate with the overall survival of lung cancer patients (Fig. 6), which implies that the M1/M2 macrophage balance in tumor microenvironment is related to lung cancer patient survival and cancer progression. Taken together, we conclude that M1/M2 macrophages had different impacts on regulation of biologic behaviors and gene expression of lung cancer cells, and M1/M2 gene expression signature may be used as a prognostic indicator for lung cancer patients. Polarizing TAMs to M1-subtype macrophages or eliminating M2-subtype macrophages, after further investigations, might represent useful anti-cancer treatment strategies in the future.

## Methods

### Cell culture and macrophage polarization

The cultures of A549 and THP-1 cells (American Type Culture Collection, Manassas, VA) are described in the Supplementary Methods. The culture supernatant that was collected from phorbol myristate acetate (PMA; Sigma, St Louis, MO)-treated THP-1 cells, named THP-1 conditioned medium (CM), was used to induce M0 macrophage differentiation. M0 macrophages were polarized into M1, M2a, or M2c macrophages, as described previously^14^ (see Supplementary Methods). For short-term cultures, A549 cells and macrophages were co-cultured (1:10) in a Transwell apparatus (0.4-μm pore size; Costar, Corning, NY) for 2 days. For long-term cultures, A549 cells were cultured in macrophage CM for at least 2 weeks.

### Flow cytometry

The expression of the cell surface markers CD68 (Darco, Copenhagen, Denmark), CD14, CD206, CD23 (BioLegend) and CCR7 (BD Bioscience Pharmingen, San Diego, CA) were used to determine the macrophage subtypes using a Cytomics FC500 flow cytometer (Beckman Coulter, Brea, CA). For the apoptosis assays, A549 cells were analyzed by flow cytometry using an annexin V-based apoptosis assay according to the manufacturer’s protocol (BD Pharmingen). For cell-cycle analyses, A549 cells were fixed by ice-cold ethanol, stained with propidium iodide, and analyzed, as described previously^44^ (see Supplementary Methods).

### Microarray analysis and real-time reverse transcription-polymerase chain reaction

For microarray experiments, A549 cells were co-cultured with or without macrophages for 2 days in a 6-well plate with Transwell apparatus (0.4-μm pore size; Costar). After incubation for 2 days, the total RNA of the cancer cells was subjected to microarray expression analysis using a Human Genome U133 Plus 2.0 GeneChip (Affymetrix, Santa Clara, CA). These array data had been uploaded into GEO (GSE50658). For real-time reverse transcription-polymerase chain reaction (RT-PCR), the expression of target genes was detected using the SYBR Green approach and the calculation was based on the relative quantitation using the comparative CT method (2^–ΔΔCT^) (see Supplementary Methods). The primers used in the microarray validation are listed in Supplementary Table 1.

### Tube-formation assays

For tube-formation assays, serum-free media that were obtained from 24-hour-cultured A549 cells (macrophage long-term–treated) were concentrated 10-fold using ultrafiltration spin columns (Millipore, Billerica MA). Human umbilical vein endothelial cells (HUVECs) were then seeded onto 96-well plates (pre-coated with Matrigel) containing concentrated media and incubated for 8 hours. The HUVEC tubule network was analyzed using the MetaXpress High Content Image Acquisition and Analysis Software (MetaXpress) (see Supplementary Methods).

### *In vivo* tumorigenesis, angiogenesis and immunohistochemical staining

Mouse experiments were approved by the Institutional Animal Care and Use Committee of National Taiwan University College of Medicine. All mice experiments were performed in accordance with relevant guidelines. Cancer cells were subcutaneously injected into NOD-SCID mice along with Matrigel (1 × 10^6^ A549 cells/mouse), and every 2–3 days, the tumor size was measured. Mice were sacrificed at 40 days, at which time the tumors were weighed and angiogenesis was assessed by immunohistochemistry using an anti-CD31 antibody (Abcam, San Francisco, CA). Microvessel numbers were scored in 3–5 randomly selected fields per tumor. For plug assays, a mixture of different macrophage subtypes and A549 cells (3:1) in Matrigel was injected into SCID mice, and after 7 days, the mice were sacrificed and angiogenesis was measured (see Supplementary Methods).

### Cell viability, proliferation, drug sensitivity and senescence assays

Cell viability was determined by counting A549 cells after 5 days of CM treatment using a trypan blue staining protocol. Cell proliferation and drug sensitivity were determined using the thiazolyl blue tetrazolium bromide (MTT) assay in a 96-well plate (VICTOR multilabel reader, PerkinElmer, Waltham, MA), according to the manufacturer’s protocol. Long-term–treated A549 cells were assayed for senescence using the Senescence β-galactosidase Staining Kit (Cell Signaling Technology, Danvers, MA), according to the manufacturer’s protocol (see Supplementary Methods).

### Statistical analysis

To evaluate the prognostic ability of the selected candidate genes, we examined their association with clinical data using a published microarray dataset that was obtained from the following four institutes: University of Michigan Cancer Center (UM), Moffitt Cancer Center (HLM), Memorial Sloan-Kettering Cancer Center (MSK) and the Dana-Farber Cancer Institute (CAN/DF)^21^. In the first step, the differentially expressed genes were selected in three groups: A549 versus M1-A549 and M2a + 2c-A549 as well as by the intersection of genes that were differentially expressed in M1-A549 versus M2a-A549 and M2c-A549. Second, the data of selected genes were collected from a published microarray dataset^21^. Third, to discover the prognosis-associated genes, univariate Cox’s proportional hazard regression analysis was performed for each candidate gene to test its association with overall survival. For those genes with a significant Cox’s regression coefficient, a risk score method was used to calculate the signature^45^. The genes that were significantly associated with overall survival were used to construct the risk score function. The risk score function was a linear combination of gene’ expressions weighted by regression coefficient from Cox’s regression. The risk score functions of M1 vs. A549, M2a/2c vs. A549, and M1 vs. M2a/2c were (−0.29 × expression level of PALM2-AKAP2) + (−0.29 × expression level of PALM2-AKAP2) + (0.06 × expression level of GPX2) + (−0.15 × expression level of LTBP4) + (0.05 × expression level of FGB) + (0.20 × expression level of HSPE1) + (−0.27 × expression level of TNIP1) + (−0.44 × expression level of HLA-C) + (−0.39 × expression level of HLA-B) + (0.11 × expression level of SCD) + (−0.28 × expression level of HLA-C) + (0.06 × expression level of FGB) + (−0.25 × expression level of C1QTNF1), (0.26 × expression level of STC2) + (0.08 × expression level of CXCL1) + (0.12 × expression level of CXCL5), and (−0.29 × expression level of PALM2-AKAP2) + (−0.29 × expression level of PALM2-AKAP2) + (0.06 × expression level of GPX2) + (0.05 × expression level of FGB) + (−0.39 × expression level of HLA-B) + (0.11 × expression level of SCD) + (−0.21 × expression level of HLA-B) + (−0.28 × expression level of HLA-C) + (0.06 × expression level of FGB) + (−0.17 × expression level of FAM129A) + (−0.16 × expression level of TNFRSF12A) + (0.06 × expression level of FGG) + (0.35 × expression level of HSPA8), respectively. The median risk score was used as the cutoff point for patient classification.

All *in vitro* experiments were performed at least in triplicate. The data are presented as the means ± standard deviations, and the significance of differences was analyzed using an analysis of variance (ANOVA) or Student’s t-test. All statistical testing was two-tailed, and *P* < 0.05 was considered statistically significant. Other statistical analyses, including those for the microarrays and survival, are described in the Supplementary Methods.

## Additional Information

**How to cite this article**: Yuan, A. *et al.* Opposite Effects of M1 and M2 Macrophage Subtypes on Lung Cancer Progression. *Sci. Rep.* **5**, 14273; doi: 10.1038/srep14273 (2015).

## Supplementary Material

Supplementary Information

## Figures and Tables

**Table 1:** Multivariate Cox regression analysis of three M1/M2 gene signatures for the overall survival of patients with NSCLC.

**Variable**	**Hazard ratio**	**95% HR C.I.**		**p-value**
**M1 vs. M2a/2c**				
Risk score	1.59	1.22	2.08	0.001
Gender	1.27	0.98	1.65	0.074
Age	1.03	1.02	1.04	<0.0001
Stage	3.51	2.60	4.74	<0.0001
**M1 vs. A549**				
Risk score	1.32	1.01	1.72	0.041
Gender	1.27	0.98	1.66	0.068
Age	1.03	1.02	1.05	<0.0001
Stage	2.12	1.81	2.48	<0.0001
**M2a/2c vs. A549**				
Risk score	1.50	1.16	1.94	0.002
Gender	1.37	1.05	1.77	0.019
Age	1.03	1.01	1.04	<0.0001
Stage	2.15	1.84	2.51	<0.0001
