# Supplementary material for: Opposite Effects of M1 and M2 Macrophage Subtypes on Lung Cancer Progression
Source: Sci Rep. 2015 Sep 24;5:14273. doi: 10.1038/srep14273 (PMC4585843; doi:10.1038/srep14273)
Supplement: Supplementary Information [file srep14273-s1.doc]

**Data Supplement:**

**Opposite Effects of M1 and M2 Macrophage Subtypes on Lung Cancer Progression**

Ang Yuan, Yi-Jing Hsiao, Hsuan-Yu Chen, Huei-Wen Chen, Chao-Chi Ho, Yu-Yun Chen, Yi-Chia Liu, Tsai-Hsia Hong, Sung-Liang Yu, Jeremy J.W. Chen, and Pan-Chyr Yang

**Correspondence:** Jeremy J.W. Chen, Institute of Biomedical Sciences, National Chung-Hsing University, No. 250, Kuo-Kuang Rd., Taichung 40227, Taiwan, ROC. Phone: 886-4-22840896 ext. 125; Fax: 886-4-22853469; Email: [jwchen@dragon.nchu.edu.tw](mailto:jwchen@dragon.nchu.edu.tw).

**1. Supplementary Methods:**

**1.1. Cell culture and macrophage polarization**

The human monocyte cell line, THP-1 (ATCC TIB 202; American Type Culture Collection, Manassas, VA), was grown in RPMI 1640 media (Invitrogen, Carlsbad, CA) supplemented with 2 g/L Na_2_HCO_3_, 4.5 g/L glucose, 2.38 g/L HEPES and 10% fetal bovine serum (FBS; Life Technologies. Grand Island, NY). The human lung cancer cell line, A549, was grown in RPMI-1640 media supplemented with 10% FBS. All cell lines were incubated at 37°C in a humidified 20% O_2_/5% CO_2_ environment. THP-1 cells were induced to differentiate into a macrophage-like phenotype by applying 3.2 × 10^-7^ M phorbol myristate acetate (PMA; Sigma, St Louis, MO) and were incubated for 24 hours. After three washes, macrophages were incubated for an additional 24 hours to eliminate the effects of PMA and then incubated in serum-free media for 24 hours. The culture supernatants were collected as macrophage-conditioned medium (CM). THP-1 cells (2 × 10^6^) were cultured in macrophage CM for 3 days and then allowed to recover by incubating them for an additional 3 days in normal media. The attached cells, which corresponded to M0 macrophages, were polarized into M1, M2a, or M2c macrophages by adding 1 μg/ml lipopolysaccharide (LPS; Sigma) plus 20 ng/ml interferon-γ (IFN-γ), 20 ng/ml interleukin-4 (IL-4), or 20 ng/ml IL-10 (BioLegend, San Diego, CA), respectively. Polarized macrophages were extensively washed to eliminate carry-over cytokines and LPS and incubated in fresh media for 24 hours, which served as the different macrophage subtype conditioned media.

**1.2. Flow cytometry**

In apoptosis assays, 1 × 10^5^ A549 cells were seeded in a 10-cm dish and cultured overnight, and the medium was then replaced with macrophage CM. After incubation for 5 days at 37°C, the cells were trypsinized, washed with PBS, and analyzed by flow cytometry using an annexin V-based apoptosis assay according to the manufacturer’s protocol (BD Pharmingen). For cell-cycle analysis, 1 × 10^6^ long-term–treated A549 cells were harvested and washed twice with ice-cold PBS, and then fixed by incubating in 100% ice-cold ethanol for 1 hour at -20°C. Cells were treated with RNase A and stained with 25 mg/ml propidium iodide. Samples were incubated for 30 minutes in the dark and analyzed by flow cytometry using the CXP Analysis software (Beckman Coulter) to define sub-G1-, S- and G2/M-phase cells.

**1.3. Real-time RT-PCR**

SYBR Green RT-PCR was performed using an ABI Prism 7900HT sequence detection system (Applied Biosystems). The expression of the target gene normalized to that of the TATA box-binding protein (TBP), which was used as an internal standard, was calculated as -ΔCT_Interest_ = -(CT_Interest_ - CT_TBP_), whereas the difference in the relative expression of the target gene between treatments was calculated using the 2^–ΔΔCT^ method. Besides, to clearly show the expression trend of gene induced in polarized macrophage subtypes compared to M0 macrophage, the base 10 logarithm of the ratio of the relative expression of gene in cytokine-treated M0 to that in untreated M0 was adopted. Here the cytokine-treated M0 represents LPS plus IFN-γ treated M0 (i.e. M1 subtype), IL-4 treated M0 (i.e. M2a subtype) and IL-10 treated M0 macrophage (i.e. M2c subtype), respectively. The log ratio greater than 0 means that the gene expression is up-regulated in the certain macrophage subtype compared to M0, and vice versa.

**1.4. Invasion and tube-formation assays**

The invasive abilities of short-term and long-term macrophage-cultured A549 cells were determined using a Transwell apparatus with Matrigel-coated membranes (BD Pharmingen). The invasion assay procedure has been described previously in detail^1^. For tube formation assays, long-term–treated A549 cells were seeded at a density of 1 × 10^6^ cells/10-cm dish in medium containing 10% FBS. After overnight culture, the medium was replaced with serum-free medium, the cells were incubated for an additional 24 hours, and the media were concentrated 10-fold using ultrafiltration spin columns (Millipore, Billerica MA). Human umbilical vein endothelial cells (HUVECs) were seeded in 96-well plates (pre-coated with Matrigel) containing concentrated media and were incubated for 8 hours. The HUVEC tubule network was measured using MetaXpress High Content Image Acquisition and Analysis Software (MetaXpress).

**1.5. *In vivo* tumorigenesis and angiogenesis**

Age-matched (~5-8 weeks) NOD/SCID mice were used for xenograft tumour models. A549 cells were co-cultured with M0, M1, M2a, or M2c macrophages for 2 days and then maintained in CM that was derived from the corresponding macrophages for 2-3 weeks. Cancer cells from each macrophage subtype co-culture were subcutaneously injected into NOD-SCID mice along with Matrigel (1 × 10^6^ A549 cells/mouse). Every 2–3 days, the tumour size was measured using calipers. The mice were sacrificed at 40 days, at which time the tumours were weighed and angiogenesis was assessed by immunohistochemistry using an anti-CD31 antibody (Abcam, San Francisco, CA). After fixing in formalin and embedding in paraffin, tumour specimens were immunohistochemically stained for CD31 and examined by microscopy. The microvessel density of tumours was calculated as the mean of three individual immunohistochemistry slides from three different mice, respectively. Each slide represents the average value of microvessel numbers of five fields.

**1.6. Cell viability, proliferation and drug sensitivity assays**

Cell viability was determined by counting A549 cells (seeded at 1 × 10^4^ cells/6-cm dish) after 5 days of CM treatment using a trypan blue staining protocol. For cell proliferation assays, A549 cells were seeded in a 96-well plate at a density of 1 × 10^3^ cells/well. The media in the wells were replaced with CM from the different macrophage subtypes, and the plates were incubated at 37°C for 1, 3 and 5 days. Independent MTT assays were performed at each time point. For the drug sensitivity assay, long-term–treated A549 cells from each incubation condition were seeded separately in a 96-well plate (1 × 10^4^ cells/well). After incubating for 3-6 hours, the medium was replaced with medium containing 2% FBS and the indicated concentrations of cisplatin, and the plate was incubated at 37°C for 2 days. The drug response was then determined using MTT assays.

**1.7. Statistical analysis**

The raw data of the microarrays were obtained according to the manufacturer’s technical manual (Affymetrix, Santa Clara, CA). To reduce the variation between experiments derived from different microarrays, the intensity values of the probes from each microarray were rescaled using a quantile normalization method. Data were filtered by a 2-fold change under false discovery rate (FDR) protection (*P* < 0.05), and the expression patterns of the selected genes were sorting by hierarchical clustering, which was performed using the GAP-Lite v0.1 software (Academia Sinica, Taipei, Taiwan), as described previously^2^. The differentially expressed genes were subjected to pathway analysis using MetaCore (GeneGo, St. Joseph, MI).

The survival curve was estimated by the Kaplan-Meier method, and the log-rank test was performed to test the difference between the survival curves. Cox proportional hazards regression analysis with the covariates age, gender and stage was used to evaluate the independent prognostic ability of each gene signature. All statistical tests were two-tailed, and *P* < 0.05 was considered statistically significant.

The platforms of our study and Shedden's study are Affymetrix U133 plus 2 and U133A, respectively. Numbers of probes of these two arrays are 54,675 and 22,215, respectively. All 22,215 probes of U133A are within U133 plus 2. In the Figure 6 A, for the (M1+M2)-associated signature, total 103 probes were identified in our data and 83 out of 103 probes can be found in the dataset of Shedden's study^3^. The number of probes with the same direction in our and Shedden's study is 13 (13/83=16%). In the Figure 6 B, for the M1-associated signature, total 87 probes were identified in our data and 72 out of 87 probes can be found in the dataset of Shedden's study. The number of probes with the same direction in our and Shedden's study is 13 (13/72=18%). In the Figure 6 C, for the M2-associated signature, total 37 probes were identified in our data and 33 out of 37 probes can be found in the dataset of Shedden's study. The number of probes with the same direction in our and Shedden's study is 3 (3/33=9%).

**2. Supplementary Table:**

Supplementary Table 1. Sequences of real-time PCR primers.

| **Genes** | **Primers sequence (5’→3’)** |
| --- | --- |
| AGR2 | Forward: GACAAGCAACAAACCCTTGATG  Reverse: AGGACAAACTGCTCTGCCAATT |
| ANG | Forward: GAAGCGGGCACCTCTAAGATAC  Reverse: CATGCCATCCTGGCCATAG |
| ANXA13 | Forward: GCAGTTACGAGCCACCTTTCA  Reverse: TGGGCACATCTCACGAGAGTT |
| AMPD3 | Forward: CAACAGTTTGTTCCTCGAATATTCC  Reverse: TCCATAAGTGCTTCCTTCGTGTAG |
| ATF3 | Forward: GTCTCTGCCTCGGAAGTGAGTG  Reverse: TGGCAAACCTCAGCTCTTCCTT |
| CCL17 | Forward: GAGCCATTCCCCTTAGAAAGCT  Reverse: CCCTGCACAGTTACAAAAACGA |
| CCL18 | Forward: GCTCTGCTGCCTCGTCTATACC  Reverse: CCGGCCTCTCTTGGTTAGG |
| CCR7 | Forward: GCTGGTGGTGGCTCTCCTT  Reverse: GTAATCGTCCGTGACCTCATCTT |
| CD163 | Forward: CAGTGCAGAAAACCCCACAA  Reverse: AAAGGATGACTGACGGGATGA |
| CD206 | Forward: GAGGGAATCTGGTCTCCATACAA  Reverse: AAGTGGAGTCCTTCATGTGATAGGT |
| CD23 | Forward: CCCGGAACGTCTCTCAAGTTT  Reverse: TCAGCTCGAAGTTCCTCCAGTT |
| CEACAM6 | Forward: TGTGCCAAGCCCATAACTCA  Reverse: GCCAGCACTCCAATCGTGAT |
| CXCL3 | Forward: TGCCAGTGCTTGCAGACACT  Reverse: GTGGCTATGACTTCGGTTTGG |
| CXCL9 | Forward: CATCTTGCTGGTTCTGATTGGA  Reverse: GTCCCTTGGTTGGTGCT |
| CXCL10 | Forward: ATTCCTGCAAGCCAATTTTGTC  Reverse: CATCTCTTCTCACCCTTCTTTTTCA |
| C15orf48 | Forward: TTCCCTTGGTGGTGTTCATGA  Reverse: CAGTTTCCCAAGGTTCTGGATT |
| ETS1 | Forward: AGCTGGCCCCAGACTTTGT  Reverse: TCCGAGGTATAGCGGGATTCT |
| FGA | Forward: GCCCTGTCAGAGACTGTGATGA  Reverse: CCTCCCAAACTGGTCTCTTGAT |
| FGB | Forward: AAAGCAGCTGCCACTCAAAAG  Reverse: TGTTGTAGCAAAGCCTCTTGCA |
| FGG | Forward: TTGGCTGGGAAATGAGAAGATT  Reverse: TGCAGTACTGGTTCTGCCATTC |
| FOSL1 | Forward: TCAGCCCGAGAACTTTTCATTC  Reverse: CCCCGAAGTCTCGGAACAT |
| G0S2 | Forward: GCCGTGCCACTAAGGTCATT  Reverse: GCACGTACAGCTTCACCATCTT |
| GADD153 | Forward: CTCTGATTGACCGAATGGTGAA  Reverse: GGGACTGATGCTCCCAATTG |
| GADD34 | Forward: ACTGCAAAGGCGGCTCAA  Reverse: CCAGACAGCCAGGAAATGGA |
| GADD45A | Forward: TCAACGTCGACCCCGATAAC  Reverse: TGTCGTTCTCGCAGCAAAAC |
| GPX2 | Forward: TTCCCTTGCAACCAATTTGG  Reverse: CCATTCACCTCACATTTTTGGA |
| ICAM1 | Forward: CCCATGAAACCGAACACACA  Reverse: GGTGTAGCTGCATGGCATATGT |
| IFI27 | Forward: GAATCGCCTCGTCCTCCATA  Reverse: AGAGTCCAGTTGCTCCCAGTGA |
| IL1B | Forward: CAGTGGCAATGAGGATGACTTG  Reverse: AGTGGTGGTCGGAGATTCGT |
| IL6 | Forward: GACAGCCACTCACCTCTTCAGA  Reverse: GTGCCTCTTTGCTGCTTTCAC |
| IL8 | Forward: ACAGCAGAGCACACAAGCTTC  Reverse: ATCAGGAAGGCTGCCAAGAG |
| IL10 | Forward: GAGGCTACGGCGCTGTCA  Reverse: TCCACGGCCTTGCTCTTG |
| IL12B | Forward: ATTCGCTCCTGCTGCTTCACA  Reverse: CGTCCAGAATAATTCTTGGCCTC |
| IL23 | Forward: ATGATGTTCCCCATATCCAGTGT  Reverse: CCGATCCTAGCAGCTTCTCATAA |
| iNOS | Forward: AGCGGGATGACTTTCCAAGA  Reverse: GGACCCCAGGCAAGATTTG |
| LAMC2 | Forward: GGCTCCTGCCAAATTTCTTG  Reverse: TGATCCGTAGACCAGCACCTT |
| MX1 | Forward: CCGCCAAGTCCAAAATTGA  Reverse: TGCAATGCACCCCTGTATACC |
| NTS | Forward: GGCTTTTCAACACTGGGAGTTAAT  Reverse: TCTCATACAGCTGCCGTTTCAG |
| PALM2-AKAP2 | Forward: CGACCTGCCAATCCTCTGTT  Reverse: GATGTTTCTGTGGCGTTCGA |
| PDZK1IP1 | Forward: TTCCTGGTCCTCGTTGCAAT  Reverse: CTGTTCCCACCAGGACTCCAT |
| PLAU | Forward: GATCCCCAGTTTGGCACAAG  Reverse: ACACTCCCGGTGGGAAATC |
| RELB | Forward: TGCTTCCAGGCCTCATATCG  Reverse: CCCGCTTTCCTTGTTAATTCG |
| SERPINB8 | Forward: CTCGCCGTGAAAGAGTGATG  Reverse: AGGGAGCATGAGACGAGAAATG |
| SERPINE1 | Forward: CGTGGTTTTCTCACCCTATGG  Reverse: ATGCCCTTGTCATCAATCTTGA |
| SESN2 | Forward: GGAGCGGAACCTCAAGGTCTA  Reverse: GCAAGTTCACGTGGACCTTCTC |
| SOD2 | Forward: TGGCCAAGGGAGATGTTACAG  Reverse: GCAACTCCCCTTTGGGTTCT |
| TGFB1 | Forward: AGGTCACCCGCGTGCTAAT  Reverse: GCTTCTCGGAGCTCTGATGTGT |
| TNFA | Forward: CTTCTGCCTGCTGCACTTTG  Reverse: GGCCAGAGGGCTGATTAGAGA |

Supplementary Table 2. Significantly differential expressed genes was validated by real-time RT-PCR.

|  |  | **Short-term culture** | | | **Long-term culture** | | | **Array** |
| --- | --- | --- | --- | --- | --- | --- | --- | --- |
| **Annotation** | **Gene name** | **M1/M0** | **M1/M2a** | **M1/M2c** | **M1/M0** | **M1/M2a** | **M1/M2c** | **M1/A549** |
| **Angiogenesis** | ANG | 0.30 | 0.42 | 0.30 | 0.49 | 0.64 | 0.40 | Down |
| **Transcriptional factor** | ATF3 | 1.64 | 2.36 | 3.69 | 5.80 | 7.22 | 52.0 | Up |
|  | ETS1 | 2.83 | 4.25 | 2.34 | 34.3 | 11.2 | 4.86 | Up |
|  | RELB | 2.91 | 1.81 | 1.14 | 38.5 | 5.16 | 9.87 | Up |
|  | FOSL1 | 1.55 | 1.09 | 1.05 | 12.8 | 3.62 | 2.02 | Up |
| **Coagulation** | PLAU | 1.48 | 2.24 | 1.34 | 3.27 | 2.43 | 4.97 | Up |
|  | SERPINB8 | - | - | - | 3.08 | 1.72 | 2.97 | Up |
|  | SERPINE1 | 1.64 | 2.73 | 1.57 | 14.5 | 3.83 | 19.6 | Up |
|  | FGB | 0.18 | 0.17 | 0.25 | 0.01 | 0.02 | 0.01 | Down |
|  | FGA | 0.28 | 0.32 | 0.45 | 0.09 | 0.05 | 0.05 | Down |
|  | FGG | 0.20 | 0.23 | 0.36 | 0.04 | 0.05 | 0.04 | Down |
| **Cell cycle** | GADD153 | 1.83 | 6.49 | 4.27 | 17.4 | 25.2 | 38.7 | Up |
|  | GADD45A | 6.32 | 3.93 | 3.37 | 9.99 | 2.56 | 16.1 | Up |
|  | SESN2 | 2.28 | 5.05 | 4.86 | 13.4 | 5.74 | 7.48 | Up |
|  | GADD34 | 2.07 | 3.59 | 2.79 | 30.9 | 24.0 | 20.3 | Up |
|  | G0S2 | 4.62 | 3.69 | 1.43 | 39.1 | 34.8 | 8.86 | Up |
| **Cell adhesion** | ICAM1 | 4.27 | 5.40 | 2.03 | 7.33 | 6.86 | 9.89 | Up |
|  | LAMC2 | 2.60 | 4.09 | 1.65 | 6.25 | 5.24 | 8.44 | Up |
|  | CEACAM6 | 0.51 | 0.41 | 0.48 | 0.03 | 0.25 | 0.01 | Down |
| **Cytokine/**  **secreted protein** | CXCL3 | 4.22 | 3.54 | 1.20 | 19.1 | 4.68 | 23.0 | Up |
|  | IL8 | 4.97 | 13.1 | 2.01 | 34.9 | 30.3 | 65.9 | Up |
|  | IL6 | 15.8 | 13.6 | 3.66 | 735 | 10.4 | 1765 | Up |
|  | AGR2 | 0.26 | 0.25 | 0.35 | 0.07 | 0.07 | 0.03 | Down |
|  | NTS | 0.33 | 0.53 | 0.47 | 0.01 | 0.04 | 0.01 | Down |
|  | TGFB1 | 0.94 | 0.54 | 0.81 | 0.03 | 0.03 | 0.03 | Down |
| **Interferon-**  **stimulation** | MX1 | 9.69 | 5.69 | 5.02 | 164 | 57.6 | 232 | Up |
|  | IFI27 | 23.9 | 17.3 | 27.7 | 3424 | 500 | 368 | Up |
| **Enzyme** | SOD2 | 3.66 | 6.36 | 1.69 | 22.5 | 13.8 | 31.7 | Up |
|  | AMPD3 | 2.48 | 2.84 | 1.57 | 68.4 | 13.2 | 7.07 | up |
|  | GPX2 | 0.38 | 0.26 | 0.40 | 0.01 | 0.04 | 0.01 | Down |
| **Unknown** | C15orf48 | 12.2 | 10.8 | 1.70 | 1594 | 7.53 | 86.9 | Up |
|  | PDZK1IP1 | 3.91 | 78.2 | 1.29 | 312 | 2.10 | 6.08 | Up |
|  | PALM2-  AKAP2 | - | - | - | 420 | 349 | 39.9 | Up |
|  | ANXA13 | 0.12 | 0.13 | 0.33 | 0.56 | 0.24 | 0.13 | Down |

- represents undetermined cycles.

Down

1.91

3.10

16.3

0.61

0.46

0.71

Inhibitor of DNA binding 1, dominant negative helix-loop-helix protein

**ID1**

Up

2.02

3.62

12.8

1.05

1.09

1.55

FOS-like antigen 1

**FOSL1**

Up

9.87

5.16

38.5

1.14

1.81

2.91

v-rel reticuloendotheliosis viral oncogene homolog B

**RELB**

Up

4.86

11.2

34.3

2.34

4.25

2.83

v-ets erythroblastosis virus E26 oncogene homolog 1 (avian)

**ETS1**

Up

52.0

7.22

5.80

3.69

2.36

1.64

Activating transcription factor 3

**ATF3**

**TF**

Down

0.40

0.64

0.49

0.30

0.42

0.30

Angiogenin, ribonuclease, RNase A family, 5

**ANG**

**Angiogenisis**

**M1/A549**

**M1/M2c**

**M1/M2a**

**M1/M0**

**M1/M2c**

**M1/M2a**

**M1/M0**

**Description**

**Gene symbol**

**Function**

**Array**

**Long-term Real-time PCR check**

**Short-term Real-time PCR check**

Down

0.13

0.24

0.56

0.33

0.13

0.12

Annexin A13

**ANXA13**

Up

39.9

349

420

－

－

－

PALM2-AKAP2 readthrough transcript

**PALM2-AKAP2**

Up

6.08

2.10

312

1.29

78.2

3.91

PDZK1 interacting protein 1

**PDZK1IP1**

Up

86.9

7.53

1594

1.70

10.8

12.2

Chromosome 15 open reading frame 48

**C15orf48**

**Unknown**

Down

0.01

0.04

0.01

0.40

0.26

0.38

Glutathione peroxidase 2 (gastrointestinal)

**GPX2**

Up

7.07

13.2

68.4

1.57

2.84

2.48

Adenosine monophosphate deaminase (isoform E)

**AMPD3**

Up

31.7

13.8

22.5

1.69

6.36

3.66

Superoxide dismutase 2, mitochondrial

**SOD2**

**Enzyme**

Up

368

500

3424

27.7

17.3

23.9

Interferon, alpha-inducible protein 27

**IFI27**

Up

232

57.6

164

5.02

5.69

9.69

Myxovirus (influenza virus) resistance 1, interferon-inducible protein p78

**MX1**

**Interferon-stimulated genes**

Down

0.69

1.55

29.2

0.21

0.20

0.35

Heat shock 70kDa protein 8

**HSPA8**

Up

0.75

0.85

1.27

2.56

2.68

2.48

Dual specificity phosphatase 6

**DUSP6**

**MAPK signaling**

Down

0.03

0.03

0.03

0.81

0.54

0.94

Transforming growth factor, beta 1

**TGFB1**

Down

0.01

0.04

0.01

0.47

0.53

0.33

Neurotensin

**NTS**

Down

0.03

0.07

0.07

0.35

0.25

0.26

Anterior gradient homolog 2

**AGR2**

Up

65.9

30.3

34.9

2.01

13.1

4.97

Interleukin 8

**IL8**

Up

23.0

4.68

19.1

1.20

3.54

4.22

Chemokine (C-X-C motif) ligand 3

**CXCL3**

**Cytokine/**

**Screted protein**

Up

81.3

18.3

40.9

0.85

0.87

1.15

Transgelin

**TAGLN**

Down

0.01

0.25

0.03

0.48

0.41

0.51

Carcinoembryonic antigen-related cell adhesion molecule 6

**CEACAM6**

Up

8.44

5.24

6.25

1.65

4.09

2.60

Laminin, gamma 2

**LAMC2**

Up

9.89

6.86

7.33

2.03

5.40

4.27

Intercellular adhesion molecule 1

**ICAM1**

**Cell adhesion**

Down

3.13

1.56

2.23

0.58

0.50

0.64

Tumor protein D52-like 1

**TPD52L1**

Up

8.86

34.8

39.1

1.43

3.69

4.62

G0/G1switch 2

**G0S2**

Up

20.3

24.0

30.9

2.79

3.59

2.07

Protein phosphatase 1, regulatory (inhibitor) subunit 15A

**PPP1R15A/**

**GADD34**

Up

7.48

5.74

13.4

4.86

5.05

2.28

Sestrin 2

**SESN2**

Up

16.1

2.56

9.99

3.37

3.93

6.32

Growth arrest and DNA-damage-inducible, alpha

**GADD45A**

Up

38.7

25.2

17.4

4.27

6.49

1.83

DNA-damage-inducible transcript 3 /// nuclear receptor subfamily 1, group H, mem

**DDIT3 /// NR1H3/**

**GADD153**

**Cell cycle**

Down

0.04

0.05

0.04

0.36

0.23

0.20

Fibrinogen gamma chain

**FGG**

Down

0.05

0.05

0.09

0.45

0.32

0.28

Fibrinogen alpha chain

**FGA**

Down

0.01

0.02

0.01

0.25

0.17

0.18

Fibrinogen beta chain

**FGB**

Up

19.6

3.83

14.5

1.57

2.73

1.64

Serpin peptidase inhibitor, clade E (nexin, plasminogen activator inhibitor type

**SERPINE1**

Up

2.97

1.72

3.08

－

－

－

Serpin peptidase inhibitor, clade B (ovalbumin), member 8

**SERPINB8**

Up

4.97

2.43

3.27

1.34

2.24

1.48

Plasminogen activator, urokinase

**PLAU**

**Coagulation**

Down

1.91

3.10

16.3

0.61

0.46

0.71

Inhibitor of DNA binding 1, dominant negative helix-loop-helix protein

**ID1**

Up

2.02

3.62

12.8

1.05

1.09

1.55

FOS-like antigen 1

**FOSL1**

Up

9.87

5.16

38.5

1.14

1.81

2.91

v-rel reticuloendotheliosis viral oncogene homolog B

**RELB**

Up

4.86

11.2

34.3

2.34

4.25

2.83

v-ets erythroblastosis virus E26 oncogene homolog 1 (avian)

**ETS1**

Up

52.0

7.22

5.80

3.69

2.36

1.64

Activating transcription factor 3

**ATF3**

**TF**

Down

0.40

0.64

0.49

0.30

0.42

0.30

Angiogenin, ribonuclease, RNase A family, 5

**ANG**

**Angiogenisis**

**M1/A549**

**M1/M2c**

**M1/M2a**

**M1/M0**

**M1/M2c**

**M1/M2a**

**M1/M0**

**Description**

**Gene symbol**

**Function**

**Array**

**Long-term Real-time PCR check**

**Short-term Real-time PCR check**

Down

0.13

0.24

0.56

0.33

0.13

0.12

Annexin A13

**ANXA13**

Up

39.9

349

420

－

－

－

PALM2-AKAP2 readthrough transcript

**PALM2-AKAP2**

Up

6.08

2.10

312

1.29

78.2

3.91

PDZK1 interacting protein 1

**PDZK1IP1**

Up

86.9

7.53

1594

1.70

10.8

12.2

Chromosome 15 open reading frame 48

**C15orf48**

**Unknown**

Down

0.01

0.04

0.01

0.40

0.26

0.38

Glutathione peroxidase 2 (gastrointestinal)

**GPX2**

Up

7.07

13.2

68.4

1.57

2.84

2.48

Adenosine monophosphate deaminase (isoform E)

**AMPD3**

Up

31.7

13.8

22.5

1.69

6.36

3.66

Superoxide dismutase 2, mitochondrial

**SOD2**

**Enzyme**

Up

368

500

3424

27.7

17.3

23.9

Interferon, alpha-inducible protein 27

**IFI27**

Up

232

57.6

164

5.02

5.69

9.69

Myxovirus (influenza virus) resistance 1, interferon-inducible protein p78

**MX1**

**Interferon-stimulated genes**

Down

0.69

1.55

29.2

0.21

0.20

0.35

Heat shock 70kDa protein 8

**HSPA8**

Up

0.75

0.85

1.27

2.56

2.68

2.48

Dual specificity phosphatase 6

**DUSP6**

**MAPK signaling**

Down

0.03

0.03

0.03

0.81

0.54

0.94

Transforming growth factor, beta 1

**TGFB1**

Down

0.01

0.04

0.01

0.47

0.53

0.33

Neurotensin

**NTS**

Down

0.03

0.07

0.07

0.35

0.25

0.26

Anterior gradient homolog 2

**AGR2**

Up

65.9

30.3

34.9

2.01

13.1

4.97

Interleukin 8

**IL8**

Up

23.0

4.68

19.1

1.20

3.54

4.22

Chemokine (C-X-C motif) ligand 3

**CXCL3**

**Cytokine/**

**Screted protein**

Up

81.3

18.3

40.9

0.85

0.87

1.15

Transgelin

**TAGLN**

Down

0.01

0.25

0.03

0.48

0.41

0.51

Carcinoembryonic antigen-related cell adhesion molecule 6

**CEACAM6**

Up

8.44

5.24

6.25

1.65

4.09

2.60

Laminin, gamma 2

**LAMC2**

Up

9.89

6.86

7.33

2.03

5.40

4.27

Intercellular adhesion molecule 1

**ICAM1**

**Cell adhesion**

Down

3.13

1.56

2.23

0.58

0.50

0.64

Tumor protein D52-like 1

**TPD52L1**

Up

8.86

34.8

39.1

1.43

3.69

4.62

G0/G1switch 2

**G0S2**

Up

20.3

24.0

30.9

2.79

3.59

2.07

Protein phosphatase 1, regulatory (inhibitor) subunit 15A

**PPP1R15A/**

**GADD34**

Up

7.48

5.74

13.4

4.86

5.05

2.28

Sestrin 2

**SESN2**

Up

16.1

2.56

9.99

3.37

3.93

6.32

Growth arrest and DNA-damage-inducible, alpha

**GADD45A**

Up

38.7

25.2

17.4

4.27

6.49

1.83

DNA-damage-inducible transcript 3 /// nuclear receptor subfamily 1, group H, mem

**DDIT3 /// NR1H3/**

**GADD153**

**Cell cycle**

Down

0.04

0.05

0.04

0.36

0.23

0.20

Fibrinogen gamma chain

**FGG**

Down

0.05

0.05

0.09

0.45

0.32

0.28

Fibrinogen alpha chain

**FGA**

Down

0.01

0.02

0.01

0.25

0.17

0.18

Fibrinogen beta chain

**FGB**

Up

19.6

3.83

14.5

1.57

2.73

1.64

Serpin peptidase inhibitor, clade E (nexin, plasminogen activator inhibitor type

**SERPINE1**

Up

2.97

1.72

3.08

－

－

－

Serpin peptidase inhibitor, clade B (ovalbumin), member 8

**SERPINB8**

Up

4.97

2.43

3.27

1.34

2.24

1.48

Plasminogen activator, urokinase

**PLAU**

**Coagulation**

Down

1.91

3.10

16.3

0.61

0.46

0.71

Inhibitor of DNA binding 1, dominant negative helix-loop-helix protein

**ID1**

Up

2.02

3.62

12.8

1.05

1.09

1.55

FOS-like antigen 1

**FOSL1**

Up

9.87

5.16

38.5

1.14

1.81

2.91

v-rel reticuloendotheliosis viral oncogene homolog B

**RELB**

Up

4.86

11.2

34.3

2.34

4.25

2.83

v-ets erythroblastosis virus E26 oncogene homolog 1 (avian)

**ETS1**

Up

52.0

7.22

5.80

3.69

2.36

1.64

Activating transcription factor 3

**ATF3**

**TF**

Down

0.40

0.64

0.49

0.30

0.42

0.30

Angiogenin, ribonuclease, RNase A family, 5

**ANG**

**Angiogenisis**

**M1/A549**

**M1/M2c**

**M1/M2a**

**M1/M0**

**M1/M2c**

**M1/M2a**

**M1/M0**

**Description**

**Gene symbol**

**Function**

**Array**

**Long-term Real-time PCR check**

**Short-term Real-time PCR check**

Down

0.13

0.24

0.56

0.33

0.13

0.12

Annexin A13

**ANXA13**

Up

39.9

349

420

－

－

－

PALM2-AKAP2 readthrough transcript

**PALM2-AKAP2**

Up

6.08

2.10

312

1.29

78.2

3.91

PDZK1 interacting protein 1

**PDZK1IP1**

Up

86.9

7.53

1594

1.70

10.8

12.2

Chromosome 15 open reading frame 48

**C15orf48**

**Unknown**

Down

0.01

0.04

0.01

0.40

0.26

0.38

Glutathione peroxidase 2 (gastrointestinal)

**GPX2**

Up

7.07

13.2

68.4

1.57

2.84

2.48

Adenosine monophosphate deaminase (isoform E)

**AMPD3**

Up

31.7

13.8

22.5

1.69

6.36

3.66

Superoxide dismutase 2, mitochondrial

**SOD2**

**Enzyme**

Up

368

500

3424

27.7

17.3

23.9

Interferon, alpha-inducible protein 27

**IFI27**

Up

232

57.6

164

5.02

5.69

9.69

Myxovirus (influenza virus) resistance 1, interferon-inducible protein p78

**MX1**

**Interferon-stimulated genes**

Down

0.69

1.55

29.2

0.21

0.20

0.35

Heat shock 70kDa protein 8

**HSPA8**

Up

0.75

0.85

1.27

2.56

2.68

2.48

Dual specificity phosphatase 6

**DUSP6**

**MAPK signaling**

Down

0.03

0.03

0.03

0.81

0.54

0.94

Transforming growth factor, beta 1

**TGFB1**

Down

0.01

0.04

0.01

0.47

0.53

0.33

Neurotensin

**NTS**

Down

0.03

0.07

0.07

0.35

0.25

0.26

Anterior gradient homolog 2

**AGR2**

Up

65.9

30.3

34.9

2.01

13.1

4.97

Interleukin 8

**IL8**

Up

23.0

4.68

19.1

1.20

3.54

4.22

Chemokine (C-X-C motif) ligand 3

**CXCL3**

**Cytokine/**

**Screted protein**

Up

81.3

18.3

40.9

0.85

0.87

1.15

Transgelin

**TAGLN**

Down

0.01

0.25

0.03

0.48

0.41

0.51

Carcinoembryonic antigen-related cell adhesion molecule 6

**CEACAM6**

Up

8.44

5.24

6.25

1.65

4.09

2.60

Laminin, gamma 2

**LAMC2**

Up

9.89

6.86

7.33

2.03

5.40

4.27

Intercellular adhesion molecule 1

**ICAM1**

**Cell adhesion**

Down

3.13

1.56

2.23

0.58

0.50

0.64

Tumor protein D52-like 1

**TPD52L1**

Up

8.86

34.8

39.1

1.43

3.69

4.62

G0/G1switch 2

**G0S2**

Up

20.3

24.0

30.9

2.79

3.59

2.07

Protein phosphatase 1, regulatory (inhibitor) subunit 15A

**PPP1R15A/**

**GADD34**

Up

7.48

5.74

13.4

4.86

5.05

2.28

Sestrin 2

**SESN2**

Up

16.1

2.56

9.99

3.37

3.93

6.32

Growth arrest and DNA-damage-inducible, alpha

**GADD45A**

Up

38.7

25.2

17.4

4.27

6.49

1.83

DNA-damage-inducible transcript 3 /// nuclear receptor subfamily 1, group H, mem

**DDIT3 /// NR1H3/**

**GADD153**

**Cell cycle**

Down

0.04

0.05

0.04

0.36

0.23

0.20

Fibrinogen gamma chain

**FGG**

Down

0.05

0.05

0.09

0.45

0.32

0.28

Fibrinogen alpha chain

**FGA**

Down

0.01

0.02

0.01

0.25

0.17

0.18

Fibrinogen beta chain

**FGB**

Up

19.6

3.83

14.5

1.57

2.73

1.64

Serpin peptidase inhibitor, clade E (nexin, plasminogen activator inhibitor type

**SERPINE1**

Up

2.97

1.72

3.08

－

－

－

Serpin peptidase inhibitor, clade B (ovalbumin), member 8

**SERPINB8**

Up

4.97

2.43

3.27

1.34

2.24

1.48

Plasminogen activator, urokinase

**PLAU**

**Coagulation**

**Table 1 Significant genes validation by Real-time PCR**

— represents undetermined cycles.

**Table 1 Significant genes validation by Real-time PCR**

— represents undetermined cycles.

Supplementary Table 3. Probe and gene list of three M1 and/or M2 gene signatures.

| **Probe ID** | **Gene name** | **Cox coefficient** | **Hazard ratio** |
| --- | --- | --- | --- |
| **M1 vs. A549^#^** |  |  |  |
| 205133_s_at | HSPE1 | 0.20 | 1.229 |
| 211708_s_at | SCD | 0.11 | 1.118 |
| 202831_at | GPX2 | 0.06 | 1.069 |
| 216238_s_at | FGB | 0.06 | 1.062 |
| 204988_at | FGB | 0.05 | 1.052 |
| 204442_x_at | LTBP4 | -0.15 | 0.860 |
| 220975_s_at | C1QTNF1 | -0.25 | 0.775 |
| 207196_s_at | TNIP1 | -0.27 | 0.759 |
| 214459_x_at | HLA-C | -0.28 | 0.755 |
| 202760_s_at | PALM2-AKAP2 | -0.29 | 0.748 |
| 202759_s_at | PALM2-AKAP2 | -0.29 | 0.742 |
| 209140_x_at | HLA-B | -0.39 | 0.670 |
| 208812_x_at | HLA-C | -0.44 | 0.642 |
| **M2a/2c vs. A549^§^** |  |  |  |
| 203439_s_at | STC2 | 0.26 | 1.302 |
| 214974_x_at | CXCL5 | 0.12 | 1.128 |
| 204470_at | CXCL1 | 0.08 | 1.093 |
| **M1 vs. M2a/2c^＊^** |  |  |  |
| 221891_x_at | HSPA8 | 0.35 | 1.431 |
| 211162_x_at | SCD | 0.11 | 1.123 |
| 219612_s_at | FGG | 0.06 | 1.066 |
| 202831_at | GPX2 | 0.06 | 1.069 |
| 216238_s_at | FGB | 0.06 | 1.062 |
| 204988_at | FGB | 0.05 | 1.052 |
| 218368_s_at | TNFRSF12A | -0.16 | 0.848 |
| 217967_s_at | FAM129A | -0.17 | 0.838 |
| 211911_x_at | HLA-B | -0.21 | 0.804 |
| 214459_x_at | HLA-C | -0.28 | 0.755 |
| 202759_s_at | PALM2-AKAP2 | -0.29 | 0.748 |
| 202760_s_at | PALM2-AKAP2 | -0.29 | 0.742 |
| 209140_x_at | HLA-B | -0.39 | 0.670 |

^#^The risk score of gene signature M1 vs. A549 = (-0.29 × expression level of PALM2-AKAP2) + (-0.29 × expression level of PALM2-AKAP2) + (0.06 × expression level of GPX2) + (-0.15 × expression level of LTBP4) + (0.05 × expression level of FGB) + (0.20 × expression level of HSPE1) + (-0.27 × expression level of TNIP1) + (-0.44 × expression level of HLA-C) + (-0.39 × expression level of HLA-B) + (0.11 × expression level of SCD) + (-0.28 × expression level of HLA-C) + (0.06 × expression level of FGB) + (-0.25 × expression level of C1QTNF1).

^§^The risk score of gene signature M2a/2c vs. A549 = (0.26 × expression level of STC2) + (0.08 × expression level of CXCL1) + (0.12 × expression level of CXCL5).

*The risk score of gene signature M1 vs. M2a/2c = (-0.29 × expression level of PALM2-AKAP2) + (-0.29 × expression level of PALM2-AKAP2) + (0.06 × expression level of GPX2) + (0.05 × expression level of FGB) + (-0.39 × expression level of HLA-B) + (0.11 × expression level of SCD) + (-0.21 × expression level of HLA-B) + (-0.28 × expression level of HLA-C) + (0.06 × expression level of FGB) + (-0.17 × expression level of FAM129A) + (-0.16 × expression level of TNFRSF12A) + (0.06 × expression level of FGG) + (0.35 × expression level of HSPA8).

**3. Supplementary Figure:**


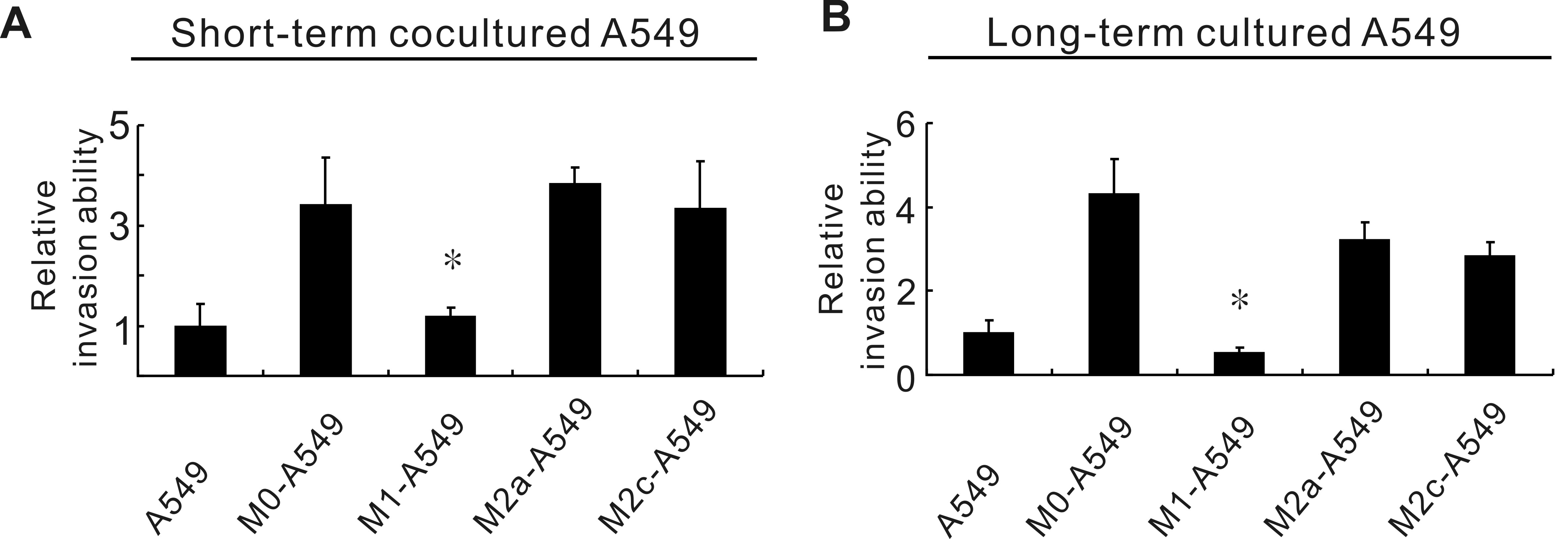


**Supplementary Figure 1. Effects of different macrophage subtypes on cancer cell invasion.** (A) Invasion ability of A549 cells after short-term co-culturing with different macrophage subtypes. (B) Invasion ability of A549 cells after long-term co-culturing with different macrophage subtypes. *P < 0.05 (mean±SD, n=3), compared to the M0-treated A549 cells.

**Supplementary References:**

1. Chen JJ, Lin YC, Yao PL, Yuan A, Chen HY, Shun CT, Tsai MF, Chen CH, Yang PC. Tumor-associated macrophages: the double-edged sword in cancer progression*. J Clin Onco*l 2005; 23: 953-964.

2. Chen CH. Generalized Association Plots for Information Visualization: The applications of the convergence of iteratively formed correlation matrices. *Statistica Sinica* 2002;12:1-23.

3. Shedden, K., et al. Gene expression-based survival prediction in lung adenocarcinoma: a multi-site, blinded validation study. Nat Med 14, 822-827 (2008).

Down

1.91

3.10

16.3

0.61

0.46

0.71

Inhibitor of DNA binding 1, dominant negative helix-loop-helix protein

**ID1**

Up

2.02

3.62

12.8

1.05

1.09

1.55

FOS-like antigen 1

**FOSL1**

Up

9.87

5.16

38.5

1.14

1.81

2.91

v-rel reticuloendotheliosis viral oncogene homolog B

**RELB**

Up

4.86

11.2

34.3

2.34

4.25

2.83

v-ets erythroblastosis virus E26 oncogene homolog 1 (avian)

**ETS1**

Up

52.0

7.22

5.80

3.69

2.36

1.64

Activating transcription factor 3

**ATF3**

**TF**

Down

0.40

0.64

0.49

0.30

0.42

0.30

Angiogenin, ribonuclease, RNase A family, 5

**ANG**

**Angiogenisis**

**M1/A549**

**M1/M2c**

**M1/M2a**

**M1/M0**

**M1/M2c**

**M1/M2a**

**M1/M0**

**Description**

**Gene symbol**

**Function**

**Array**

**Long-term Real-time PCR check**

**Short-term Real-time PCR check**

Down

0.13

0.24

0.56

0.33

0.13

0.12

Annexin A13

**ANXA13**

Up

39.9

349

420

－

－

－

PALM2-AKAP2 readthrough transcript

**PALM2-AKAP2**

Up

6.08

2.10

312

1.29

78.2

3.91

PDZK1 interacting protein 1

**PDZK1IP1**

Up

86.9

7.53

1594

1.70

10.8

12.2

Chromosome 15 open reading frame 48

**C15orf48**

**Unknown**

Down

0.01

0.04

0.01

0.40

0.26

0.38

Glutathione peroxidase 2 (gastrointestinal)

**GPX2**

Up

7.07

13.2

68.4

1.57

2.84

2.48

Adenosine monophosphate deaminase (isoform E)

**AMPD3**

Up

31.7

13.8

22.5

1.69

6.36

3.66

Superoxide dismutase 2, mitochondrial

**SOD2**

**Enzyme**

Up

368

500

3424

27.7

17.3

23.9

Interferon, alpha-inducible protein 27

**IFI27**

Up

232

57.6

164

5.02

5.69

9.69

Myxovirus (influenza virus) resistance 1, interferon-inducible protein p78

**MX1**

**Interferon-stimulated genes**

Down

0.69

1.55

29.2

0.21

0.20

0.35

Heat shock 70kDa protein 8

**HSPA8**

Up

0.75

0.85

1.27

2.56

2.68

2.48

Dual specificity phosphatase 6

**DUSP6**

**MAPK signaling**

Down

0.03

0.03

0.03

0.81

0.54

0.94

Transforming growth factor, beta 1

**TGFB1**

Down

0.01

0.04

0.01

0.47

0.53

0.33

Neurotensin

**NTS**

Down

0.03

0.07

0.07

0.35

0.25

0.26

Anterior gradient homolog 2

**AGR2**

Up

65.9

30.3

34.9

2.01

13.1

4.97

Interleukin 8

**IL8**

Up

23.0

4.68

19.1

1.20

3.54

4.22

Chemokine (C-X-C motif) ligand 3

**CXCL3**

**Cytokine/**

**Screted protein**

Up

81.3

18.3

40.9

0.85

0.87

1.15

Transgelin

**TAGLN**

Down

0.01

0.25

0.03

0.48

0.41

0.51

Carcinoembryonic antigen-related cell adhesion molecule 6

**CEACAM6**

Up

8.44

5.24

6.25

1.65

4.09

2.60

Laminin, gamma 2

**LAMC2**

Up

9.89

6.86

7.33

2.03

5.40

4.27

Intercellular adhesion molecule 1

**ICAM1**

**Cell adhesion**

Down

3.13

1.56

2.23

0.58

0.50

0.64

Tumor protein D52-like 1

**TPD52L1**

Up

8.86

34.8

39.1

1.43

3.69

4.62

G0/G1switch 2

**G0S2**

Up

20.3

24.0

30.9

2.79

3.59

2.07

Protein phosphatase 1, regulatory (inhibitor) subunit 15A

**PPP1R15A/**

**GADD34**

Up

7.48

5.74

13.4

4.86

5.05

2.28

Sestrin 2

**SESN2**

Up

16.1

2.56

9.99

3.37

3.93

6.32

Growth arrest and DNA-damage-inducible, alpha

**GADD45A**

Up

38.7

25.2

17.4

4.27

6.49

1.83

DNA-damage-inducible transcript 3 /// nuclear receptor subfamily 1, group H, mem

**DDIT3 /// NR1H3/**

**GADD153**

**Cell cycle**

Down

0.04

0.05

0.04

0.36

0.23

0.20

Fibrinogen gamma chain

**FGG**

Down

0.05

0.05

0.09

0.45

0.32

0.28

Fibrinogen alpha chain

**FGA**

Down

0.01

0.02

0.01

0.25

0.17

0.18

Fibrinogen beta chain

**FGB**

Up

19.6

3.83

14.5

1.57

2.73

1.64

Serpin peptidase inhibitor, clade E (nexin, plasminogen activator inhibitor type

**SERPINE1**

Up

2.97

1.72

3.08

－

－

－

Serpin peptidase inhibitor, clade B (ovalbumin), member 8

**SERPINB8**

Up

4.97

2.43

3.27

1.34

2.24

1.48

Plasminogen activator, urokinase

**PLAU**

**Coagulation**

Down

1.91

3.10

16.3

0.61

0.46

0.71

Inhibitor of DNA binding 1, dominant negative helix-loop-helix protein

**ID1**

Up

2.02

3.62

12.8

1.05

1.09

1.55

FOS-like antigen 1

**FOSL1**

Up

9.87

5.16

38.5

1.14

1.81

2.91

v-rel reticuloendotheliosis viral oncogene homolog B

**RELB**

Up

4.86

11.2

34.3

2.34

4.25

2.83

v-ets erythroblastosis virus E26 oncogene homolog 1 (avian)

**ETS1**

Up

52.0

7.22

5.80

3.69

2.36

1.64

Activating transcription factor 3

**ATF3**

**TF**

Down

0.40

0.64

0.49

0.30

0.42

0.30

Angiogenin, ribonuclease, RNase A family, 5

**ANG**

**Angiogenisis**

**M1/A549**

**M1/M2c**

**M1/M2a**

**M1/M0**

**M1/M2c**

**M1/M2a**

**M1/M0**

**Description**

**Gene symbol**

**Function**

**Array**

**Long-term Real-time PCR check**

**Short-term Real-time PCR check**

Down

0.13

0.24

0.56

0.33

0.13

0.12

Annexin A13

**ANXA13**

Up

39.9

349

420

－

－

－

PALM2-AKAP2 readthrough transcript

**PALM2-AKAP2**

Up

6.08

2.10

312

1.29

78.2

3.91

PDZK1 interacting protein 1

**PDZK1IP1**

Up

86.9

7.53

1594

1.70

10.8

12.2

Chromosome 15 open reading frame 48

**C15orf48**

**Unknown**

Down

0.01

0.04

0.01

0.40

0.26

0.38

Glutathione peroxidase 2 (gastrointestinal)

**GPX2**

Up

7.07

13.2

68.4

1.57

2.84

2.48

Adenosine monophosphate deaminase (isoform E)

**AMPD3**

Up

31.7

13.8

22.5

1.69

6.36

3.66

Superoxide dismutase 2, mitochondrial

**SOD2**

**Enzyme**

Up

368

500

3424

27.7

17.3

23.9

Interferon, alpha-inducible protein 27

**IFI27**

Up

232

57.6

164

5.02

5.69

9.69

Myxovirus (influenza virus) resistance 1, interferon-inducible protein p78

**MX1**

**Interferon-stimulated genes**

Down

0.69

1.55

29.2

0.21

0.20

0.35

Heat shock 70kDa protein 8

**HSPA8**

Up

0.75

0.85

1.27

2.56

2.68

2.48

Dual specificity phosphatase 6

**DUSP6**

**MAPK signaling**

Down

0.03

0.03

0.03

0.81

0.54

0.94

Transforming growth factor, beta 1

**TGFB1**

Down

0.01

0.04

0.01

0.47

0.53

0.33

Neurotensin

**NTS**

Down

0.03

0.07

0.07

0.35

0.25

0.26

Anterior gradient homolog 2

**AGR2**

Up

65.9

30.3

34.9

2.01

13.1

4.97

Interleukin 8

**IL8**

Up

23.0

4.68

19.1

1.20

3.54

4.22

Chemokine (C-X-C motif) ligand 3

**CXCL3**

**Cytokine/**

**Screted protein**

Up

81.3

18.3

40.9

0.85

0.87

1.15

Transgelin

**TAGLN**

Down

0.01

0.25

0.03

0.48

0.41

0.51

Carcinoembryonic antigen-related cell adhesion molecule 6

**CEACAM6**

Up

8.44

5.24

6.25

1.65

4.09

2.60

Laminin, gamma 2

**LAMC2**

Up

9.89

6.86

7.33

2.03

5.40

4.27

Intercellular adhesion molecule 1

**ICAM1**

**Cell adhesion**

Down

3.13

1.56

2.23

0.58

0.50

0.64

Tumor protein D52-like 1

**TPD52L1**

Up

8.86

34.8

39.1

1.43

3.69

4.62

G0/G1switch 2

**G0S2**

Up

20.3

24.0

30.9

2.79

3.59

2.07

Protein phosphatase 1, regulatory (inhibitor) subunit 15A

**PPP1R15A/**

**GADD34**

Up

7.48

5.74

13.4

4.86

5.05

2.28

Sestrin 2

**SESN2**

Up

16.1

2.56

9.99

3.37

3.93

6.32

Growth arrest and DNA-damage-inducible, alpha

**GADD45A**

Up

38.7

25.2

17.4

4.27

6.49

1.83

DNA-damage-inducible transcript 3 /// nuclear receptor subfamily 1, group H, mem

**DDIT3 /// NR1H3/**

**GADD153**

**Cell cycle**

Down

0.04

0.05

0.04

0.36

0.23

0.20

Fibrinogen gamma chain

**FGG**

Down

0.05

0.05

0.09

0.45

0.32

0.28

Fibrinogen alpha chain

**FGA**

Down

0.01

0.02

0.01

0.25

0.17

0.18

Fibrinogen beta chain

**FGB**

Up

19.6

3.83

14.5

1.57

2.73

1.64

Serpin peptidase inhibitor, clade E (nexin, plasminogen activator inhibitor type

**SERPINE1**

Up

2.97

1.72

3.08

－

－

－

Serpin peptidase inhibitor, clade B (ovalbumin), member 8

**SERPINB8**

Up

4.97

2.43

3.27

1.34

2.24

1.48

Plasminogen activator, urokinase

**PLAU**

**Coagulation**

Down

1.91

3.10

16.3

0.61

0.46

0.71

Inhibitor of DNA binding 1, dominant negative helix-loop-helix protein

**ID1**

Up

2.02

3.62

12.8

1.05

1.09

1.55

FOS-like antigen 1

**FOSL1**

Up

9.87

5.16

38.5

1.14

1.81

2.91

v-rel reticuloendotheliosis viral oncogene homolog B

**RELB**

Up

4.86

11.2

34.3

2.34

4.25

2.83

v-ets erythroblastosis virus E26 oncogene homolog 1 (avian)

**ETS1**

Up

52.0

7.22

5.80

3.69

2.36

1.64

Activating transcription factor 3

**ATF3**

**TF**

Down

0.40

0.64

0.49

0.30

0.42

0.30

Angiogenin, ribonuclease, RNase A family, 5

**ANG**

**Angiogenisis**

**M1/A549**

**M1/M2c**

**M1/M2a**

**M1/M0**

**M1/M2c**

**M1/M2a**

**M1/M0**

**Description**

**Gene symbol**

**Function**

**Array**

**Long-term Real-time PCR check**

**Short-term Real-time PCR check**

Down

0.13

0.24

0.56

0.33

0.13

0.12

Annexin A13

**ANXA13**

Up

39.9

349

420

－

－

－

PALM2-AKAP2 readthrough transcript

**PALM2-AKAP2**

Up

6.08

2.10

312

1.29

78.2

3.91

PDZK1 interacting protein 1

**PDZK1IP1**

Up

86.9

7.53

1594

1.70

10.8

12.2

Chromosome 15 open reading frame 48

**C15orf48**

**Unknown**

Down

0.01

0.04

0.01

0.40

0.26

0.38

Glutathione peroxidase 2 (gastrointestinal)

**GPX2**

Up

7.07

13.2

68.4

1.57

2.84

2.48

Adenosine monophosphate deaminase (isoform E)

**AMPD3**

Up

31.7

13.8

22.5

1.69

6.36

3.66

Superoxide dismutase 2, mitochondrial

**SOD2**

**Enzyme**

Up

368

500

3424

27.7

17.3

23.9

Interferon, alpha-inducible protein 27

**IFI27**

Up

232

57.6

164

5.02

5.69

9.69

Myxovirus (influenza virus) resistance 1, interferon-inducible protein p78

**MX1**

**Interferon-stimulated genes**

Down

0.69

1.55

29.2

0.21

0.20

0.35

Heat shock 70kDa protein 8

**HSPA8**

Up

0.75

0.85

1.27

2.56

2.68

2.48

Dual specificity phosphatase 6

**DUSP6**

**MAPK signaling**

Down

0.03

0.03

0.03

0.81

0.54

0.94

Transforming growth factor, beta 1

**TGFB1**

Down

0.01

0.04

0.01

0.47

0.53

0.33

Neurotensin

**NTS**

Down

0.03

0.07

0.07

0.35

0.25

0.26

Anterior gradient homolog 2

**AGR2**

Up

65.9

30.3

34.9

2.01

13.1

4.97

Interleukin 8

**IL8**

Up

23.0

4.68

19.1

1.20

3.54

4.22

Chemokine (C-X-C motif) ligand 3

**CXCL3**

**Cytokine/**

**ScretedSecreted protein**

Up

81.3

18.3

40.9

0.85

0.87

1.15

Transgelin

**TAGLN**

Down

0.01

0.25

0.03

0.48

0.41

0.51

Carcinoembryonic antigen-related cell adhesion molecule 6

**CEACAM6**

Up

8.44

5.24

6.25

1.65

4.09

2.60

Laminin, gamma 2

**LAMC2**

Up

9.89

6.86

7.33

2.03

5.40

4.27

Intercellular adhesion molecule 1

**ICAM1**

**Cell adhesion**

Down

3.13

1.56

2.23

0.58

0.50

0.64

Tumor protein D52-like 1

**TPD52L1**

Up

8.86

34.8

39.1

1.43

3.69

4.62

G0/G1switch 2

**G0S2**

Up

20.3

24.0

30.9

2.79

3.59

2.07

Protein phosphatase 1, regulatory (inhibitor) subunit 15A

**PPP1R15A/**

**GADD34**

Up

7.48

5.74

13.4

4.86

5.05

2.28

Sestrin 2

**SESN2**

Up

16.1

2.56

9.99

3.37

3.93

6.32

Growth arrest and DNA-damage-inducible, alpha

**GADD45A**

Up

38.7

25.2

17.4

4.27

6.49

1.83

DNA-damage-inducible transcript 3 /// nuclear receptor subfamily 1, group H, mem

**DDIT3 /// NR1H3/**

**GADD153**

**Cell cycle**

Down

0.04

0.05

0.04

0.36

0.23

0.20

Fibrinogen gamma chain

**FGG**

Down

0.05

0.05

0.09

0.45

0.32

0.28

Fibrinogen alpha chain

**FGA**

Down

0.01

0.02

0.01

0.25

0.17

0.18

Fibrinogen beta chain

**FGB**

Up

19.6

3.83

14.5

1.57

2.73

1.64

Serpin peptidase inhibitor, clade E (nexin, plasminogen activator inhibitor type

**SERPINE1**

Up

2.97

1.72

3.08

－

－

－

Serpin peptidase inhibitor, clade B (ovalbumin), member 8

**SERPINB8**

Up

4.97

2.43

3.27

1.34

2.24

1.48

Plasminogen activator, urokinase

**PLAU**

**Coagulation**

**Table 1 Significant genes validation by Real-time PCR**

— represents undetermined cycles.

**Table 1 Significant genes validation by Real-time PCR**

— represents undetermined cycles.
